# A High-Throughput Screening Approach to Discovering Good Forms of Biologically Inspired Visual Representation

**DOI:** 10.1371/journal.pcbi.1000579

**Published:** 2009-11-26

**Authors:** Nicolas Pinto, David Doukhan, James J. DiCarlo, David D. Cox

**Affiliations:** 1 McGovern Institute for Brain Research, Massachusetts Institute of Technology, Cambridge, Massachussetts, United States of America; 2 Department of Brain and Cognitive Sciences, Massachusetts Institute of Technology, Cambridge, Massachussetts, United States of America; 3 The Rowland Institute at Harvard, Harvard University, Cambridge, Massachusetts, United States of America; University College London, United Kingdom

## Abstract

While many models of biological object recognition share a common set of “broad-stroke” properties, the performance of any one model depends strongly on the choice of parameters in a particular instantiation of that model—e.g., the number of units per layer, the size of pooling kernels, exponents in normalization operations, etc. Since the number of such parameters (explicit or implicit) is typically large and the computational cost of evaluating one particular parameter set is high, the space of possible model instantiations goes largely unexplored. Thus, when a model fails to approach the abilities of biological visual systems, we are left uncertain whether this failure is because we are missing a fundamental idea or because the correct “parts” have not been tuned correctly, assembled at sufficient scale, or provided with enough training. Here, we present a high-throughput approach to the exploration of such parameter sets, leveraging recent advances in stream processing hardware (high-end NVIDIA graphic cards and the PlayStation 3's IBM Cell Processor). In analogy to high-throughput screening approaches in molecular biology and genetics, we explored thousands of potential network architectures and parameter instantiations, screening those that show promising object recognition performance for further analysis. We show that this approach can yield significant, reproducible gains in performance across an array of basic object recognition tasks, consistently outperforming a variety of state-of-the-art purpose-built vision systems from the literature. As the scale of available computational power continues to expand, we argue that this approach has the potential to greatly accelerate progress in both artificial vision and our understanding of the computational underpinning of biological vision.

## Author Summary

One of the primary obstacles to understanding the computational underpinnings of biological vision is its sheer scale—the visual system is a massively parallel computer, comprised of billions of elements. While this scale has historically been beyond the reach of even the fastest super-computing systems, recent advances in commodity graphics processors (such as those found in the PlayStation 3 and high-end NVIDIA graphics cards) have made unprecedented computational resources broadly available. Here, we describe a high-throughput approach that harnesses the power of modern graphics hardware to search a vast space of large-scale, biologically inspired candidate models of the visual system. The best of these models, drawn from thousands of candidates, outperformed a variety of state-of-the-art vision systems across a range of object and face recognition tasks. We argue that these experiments point a new way forward, both in the creation of machine vision systems and in providing insights into the computational underpinnings of biological vision.

## Introduction

The study of biological vision and the creation of artificial vision systems are naturally intertwined—exploration of the neuronal substrates of visual processing provides clues and inspiration for artificial systems, and artificial systems, in turn, serve as important generators of new ideas and working hypotheses. The results of this synergy have been powerful: in addition to providing important theoretical frameworks for empirical investigations (e.g. [1]–[6]), biologically-inspired models are routinely among the highest-performing artificial vision systems in practical tests of object and face recognition [7]–[12].

However, while neuroscience has provided inspiration for some of the “broad-stroke” properties of the visual system, much is still unknown. Even for those qualitative properties that most biologically-inspired models share, experimental data currently provide little constraint on their key parameters. As a result, even the most faithfully biomimetic vision models necessarily represent just one of many possible realizations of a collection of computational ideas.

Truly evaluating the set of biologically-inspired computational ideas is difficult, since the performance of a model depends strongly on its particular instantiation–the size of the pooling kernels, the number of units per layer, exponents in normalization operations, etc. Because the number of such parameters (explicit or implicit) is typically large, and the computational cost of evaluating one particular model is high, it is difficult to adequately explore the space of possible model instantiations. At the same time, there is no guarantee that even the “correct” set of principles will work when instantiated on a small scale (in terms of dimensionality, amount of training, etc.). Thus, when a model fails to approach the abilities of biological visual systems, we cannot tell if this is because the ideas are wrong, or they are simply not put together correctly or on a large enough scale.

As a result of these factors, the availability of computational resources plays a critical role in shaping what kinds of computational investigations are possible. Traditionally, this bound has grown according to Moore's Law [13], however, recently, advances in highly-parallel graphics processing hardware (such as high-end NVIDIA graphics cards, and the PlayStation 3's IBM Cell processor) have disrupted this status quo for some classes of computational problems. In particular, this new class of modern graphics processing hardware has enabled over hundred-fold speed-ups in some of the key computations that most biologically-inspired visual models share in common. As is already occurring in other scientific fields [14],[15], the large quantitative performance improvements offered by this new class of hardware hold the potential to effect qualitative changes in how science is done.

In the present work, we take advantage of these recent advances in graphics processing hardware [16],[17] to more expansively explore the range of biologically-inspired models–including models of larger, more realistic scale. In analogy to high-throughput screening approaches in molecular biology and genetics, we generated and trained thousands of potential network architectures and parameter instantiations, and we “screened” the visual representations produced by these models using tasks that engage the core problem of object recognition–tolerance to image variation [10]–[12],[18],[19]. From these candidate models, the most promising were selected for further analysis.

We show that this large-scale screening approach can yield significant, reproducible gains in performance in a variety of basic object recognitions tasks and that it holds the promise of offering insight into which computational ideas are most important for achieving this performance. Critically, such insights can then be fed back into the design of candidate models (constraining the search space and suggesting additional model features), further guiding evolutionary progress. As the scale of available computational power continues to expand, high-throughput exploration of ideas in computational vision holds great potential both for accelerating progress in artificial vision, and for generating new, experimentally-testable hypotheses for the study of biological vision.

## Methods

### A Family of Candidate Models

In order to generate a large number of candidate model instantiations, it is necessary to parameterize the family of all possible models that will be considered. A schematic of the overall architecture of this model family, and some of its parameters, is shown in Figure 2. The parameterization of this family of models was designed to be as inclusive as possible–that is, the set of model operations and parameters was chosen so that the family of possible models would encompass (as special cases) many of the biologically-inspired models already described in the extant literature (e.g. [1]–[4],[7],[9]). For instance, the full model includes an optional “trace” term, which allows learning behavior akin to that described in previous work (e.g. [4], [20]–[22]). While some of the variation within this family of possible models might best be described as variation in parameter tuning within a fixed model architecture, many parameters produce significant architectural changes in the model (e.g. number of filters in each layer). The primary purpose of this report is to present an overarching approach to high-throughput screening. While precise choices of parameters and parameter ranges are clearly important, one could change which parameters were explored, and over what ranges, without disrupting the integrity of the overarching approach. An exhaustive description of specific model parameters used here is included in the Supplemental Text S1, and is briefly described next.

**Figure 1:**
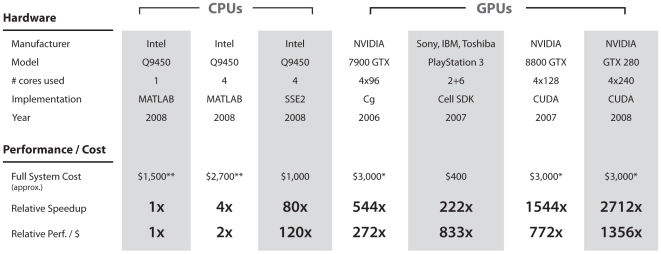
Performance and cost of various CPU and GPU implementations of a critical component of our model family. Our implemented performance speed-ups for a key filtering operation in our biologically-inspired model implementation. Performance and price are shown across a collection of different GPUs, relative to a commonly used MATLAB CPU-based implementation (using a single CPU core with the *filter2* function, which is coded in C++). We contrast this standard implementation with a multi-core MATLAB version, a highly-optimized C/SSE2 multi-core implementation on the same CPU, and highly-optimized GPU implementations. We have implemented speedups of over thousands of times with GPUs, resulting in qualitative changes in what kinds of model investigations are possible. More technical details and a throughout discussion of the computational framework enabling these speedups can be found in Supplemental Figure S1 and Supplemental Text S2. * These costs are based on multi-GPU systems containing four GPUs in addition to the quad-core CPU (Q9450). ** These costs include both the hardware and MATLAB yearly licenses (based on an academic discount pricing, for one year).

Model parameters were organized into four basic groups. The first group of parameters controlled structural properties of the system, such as the number of filters in each layer and their sizes. The second group of parameters controlled the properties of nonlinearities within each layer, such as divisive normalization coeffients and activation functions. The third group of parameters controlled how the models learned filter weights in response to video inputs during an *Unsupervised Learning Phase* (this class includes parameters such as learning rate, trace factors, etc.; see *Phase 2: Unsupervised Learning* below). A final set of parameters controlled details of how the resulting representation vectors are classified during screening and validation (e.g. parameters of dimensionality reduction, classification parameters, etc.). For the purposes of the work presented here, this class of classification-related parameters was held constant for all analyses below. Briefly, the output values of the final model layer corresponding to each test example image were “unrolled” into a vector, their dimensionality was reduced using Principal Component Analysis (PCA) keeping as many dimensions as there were data points in the training set, and labeled examples were used to train a linear Support Vector Machine (SVM).

Each model consisted of three layers, with each layer consisting of a “stack” of between 16 and 256 linear filters that were applied at each position to a region of the layer below. At each stage, the output of each unit was normalized by the activity of its neighbors within a parametrically-defined radius. Unit outputs were also subject to parameterized threshold and saturation functions, and the output of a given layer could be spatially resampled before being given to the next layer as input. Filter kernels within each stack within each layer were initialized to random starting values, and learned their weights during the *Unsupervised Learning Phase* (see below, see Supplemental Text S1). Briefly, during this phase, under parametric control, a “winning” filter or filters were selected for each input patch, and the kernel of these filters was adapted to more closely resemble that patch, achieving a form of online non-parametric density estimation. Building upon recent findings from visual neuroscience [18],[23],[24], unsupervised learning could also be biased by temporal factors, such that filters that “won” in previous frames were biased to win again (see Supplemental Text S1 for details).

It should be noted that while the parameter set describing the model family is large, it is not without constraints. While our model family includes a wide variety of feed-forward architectures with local intrinsic processing (normalization), we have not yet included long-range feedback mechanisms (e.g. layer to layer). While such mechanisms may very well turn out to be critically important for achieving the performance of natural visual systems, the intent of the current work is to present a framework to approach the problem. Other parameters and mechanisms could be added to this framework, without loss of generality. Indeed, the addition of new mechanisms and refinement of existing ones is a major area for future research (see Discussion).

### Parallel Computing Using Commodity Graphics Hardware

While details of the implementation of our model class are not essential to the theoretical implications of our approach, attention must nonetheless be paid to speed in order to ensure the practical tractability, since the models used here are large (i.e. they have many units), and because the space of possible models is enormous. Fortunately, the computations underlying our particular family of candidate models are intrinsically parallel at a number of levels. In addition to coarse-grain parallelism at the level of individual model instantiations (e.g. multiple models can be evaluated at the same time) and video frames (e.g. feedforward processing can be done in parallel on multiple frames at once), there is a high degree of fine-grained parallelism in the processing of each individual frame. For instance, when a filter kernel is applied to an image, the same filter is applied to many regions of the image, and many filters are applied to each region of the image, and these operations are largely independent. The large number of arithmetic operations per region of image also results in high arithmetic intensity (numbers of arithmetic operations per memory fetch), which is desirable for high-performance computing, since memory accesses are typically several orders of magnitude less efficient than arithmetic operations (when arithmetic intensity is high, caching of fetched results leads to better utilization of a processor's compute resources). These considerations are especially important for making use of modern graphics hardware (such as the Cell processor and GPUs) where many processors are available. Highly-optimized implementations of core operations (e.g. linear filtering, local normalization) were created for both the IBM Cell Processor (PlayStation 3), and for NVIDIA graphics processing units (GPUs) using the Tesla Architecture and the CUDA programming model [25]. These implementations achieve highly significant speed-ups relative to conventional CPU-based implementations (see Figure 1 and Supplemental Figure S1). High-level “outer loop” coordination of these highly optimized operations was accomplished using the Python programming language (e.g. using PyCUDA [26]), allowing for a favorable balance between ease of programming and raw speed (see Supplemental Text S2). In principle, all of the analyses presented here could have been performed using traditional computational hardware; however, the cost (in terms of time and/or money) of doing so with current CPU hardware is prohibitive.

**Figure 2:**
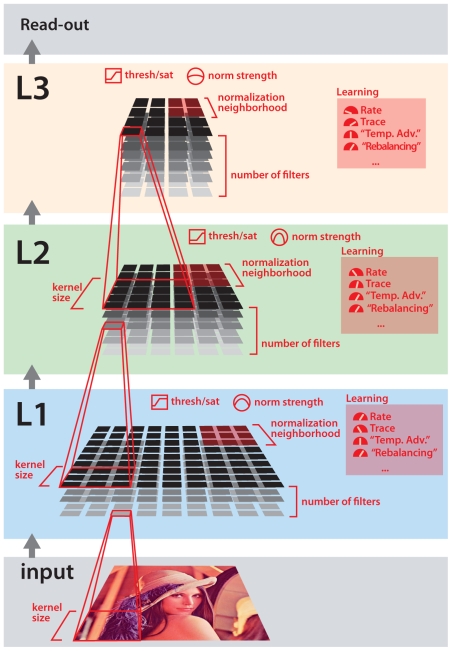
A schematic diagram of the system architecture of the family of models considered. The system consists of three feedforward filtering layers, with the filters in each layer being applied across the previous layer. Red colored labels indicate a selection of configurable parameters (only a subset of parameters are shown).

Figure 1 shows the relative speedup and performance/cost of each implementation (IBM Cell on Sony's PlayStation 3 and several NVIDIA GPUs) relative to traditional MATLAB and multi-threaded C code for the linear filtering operation (more details such as the raw floating point performance can be found in the Supplemental Figure S1). This operation is not only a key component of the candidate model family (see below) but it's also the most computationally demanding, reaching up to 94% of the total processing time (for the PlayStation 3 implementation), depending on model parameters (average fraction is 28%). The use of commodity graphics hardware affords orders-of-magnitude increases in performance. In particular, it should be noted that the data presented in this work took approximately one week to generate using our PlayStation 3-based implementation (222x speedup with one system) on a cluster of 23 machines. We estimate that producing the same results at the same cost using a conventional MATLAB implementation would have taken more than two years (see Figure S1).

### Screening for Good Forms of Representation

**Figure 3:**
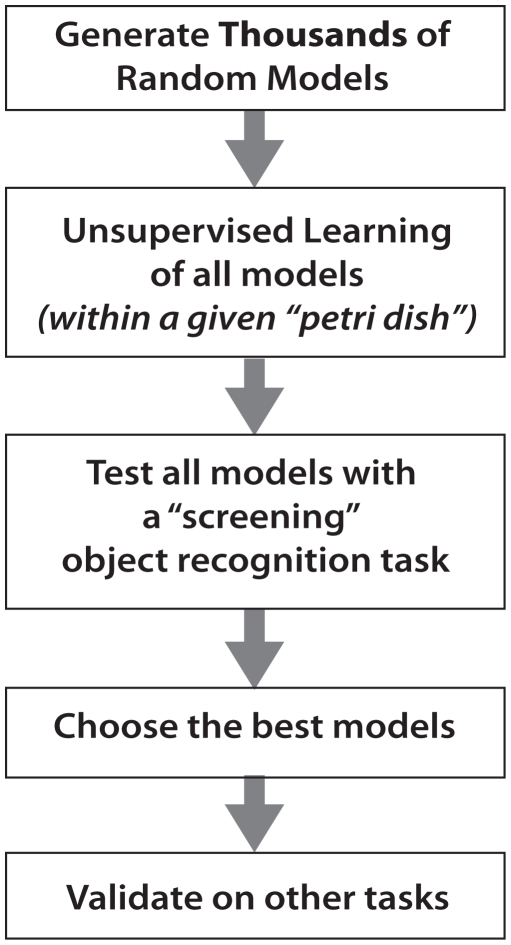
Experimental flow. The experiments described here consist of five phases. (A) First, a large collection of model instantiations are generated with randomly selected parameter values. (B) Each of these models then undergoes an unsupervised learning period, during which its filter kernels are adapted to spatio-temporal statistics of the video inputs, using a learning algorithm that is influenced by the particular parameter instantiation of that model. After the *Unsupervised Learning Phase* is complete, filter kernels are fixed, and (C) each model is subjected to a screening object recognition test, where labeled images are represented using each model instantiation, and these re-represented images are used to train an SVM to perform a simple two-class discrimination task. Performance of each candidate model is assessed using a standard cross-validation procedure. (D) From all of the model instantiations, the best are selected for further analysis. (E) Finally, these models are tested on other object recognition tasks.

Our approach is to sample a large number of model instantiations, using a well-chosen “screening” task to find promising architectures and parameter ranges within the model family. Our approach to this search was divided into four phases (see Figure 3): Candidate Model Generation, Unsupervised Learning, Screening, and Validation/Analysis of high-performing models.

#### Phase 1: candidate model generation

Candidate model parameter sets were randomly sampled with a uniform distribution from the full space of possible models in the family considered here (see Figure 2 and Figure S2 for a schematic diagram of the models, and Supplemental Materials for an exhaustive description of model parameters and value ranges that were explored; Supplemental Text S1).

#### Phase 2: unsupervised learning

All models were subjected to a period of unsupervised learning, during which filter kernels were adapted to spatiotemporal statistics of a stream of input images. Since the family of models considered here includes features designed to take advantage of the temporal statistics of natural inputs (see Supplementary Methods), models were learned using video data. In the current version of our family of models, learning influenced the form of the linear kernels of units at each layer of the hierarchy, but did not influence any other parameters of the model.

We used three video sets for unsupervised learning: “Cars and Planes”, “Boats”, and “Law and Order”. The “Law and Order” video set consisted of clips from the television program of the same name (Copyright NBC Universal), taken from DVDs, with clips selected to avoid the inclusion of text subtitles. These clips included a variety of objects moving through the frame, including characters' bodies and faces.

**Figure 4:**
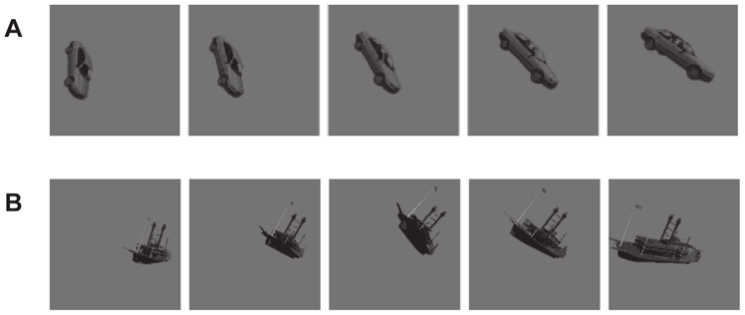
Example video frames used as input during the *Unsupervised Learning Phase*. (A) Sequences of a rendered car undergoing a random walk through the possible range of rigid body movements. (B) A similar random walk with a rendered boat.

The “Cars and Planes” and “Boats” video sets consisted of 3D ray-traced cars, planes and boats undergoing 6-degree-of-freedom view transformations (roughly speaking, “tumbling” through space). These same 3D models were also used in a previous study [11]. Video clips were generated where an object would appear for approximately 300 frames, performing a random walk in position (3 degrees of freedom) and rotation (3 degrees of freedom) for a total of 15,000 frames. Examples are shown in Figures 4A and 4B.

For the sake of convenience, we refer to each unsupervised learning video set as a “petri dish,” carrying forward the analogy to high-throughput screening from biology. In the results presented here, 2,500 model instantiations were independently generated in each “petri dish” by randomly drawing parameter values from a uniform distribution (a total of 7,500 models were trained). Examples of filter kernels resulting from this unsupervised learning procedure are shown in Supplemental Figures S3, S4, S5 and S6.

After the end of the *Unsupervised Learning Phase*, the linear filter kernels were not modified further, and the resulting model was treated as a fixed transformation (e.g. a static image is entered as input, and a vector of responses from the units of the final layer is outputted).

#### Phase 3: screening

Following the *Unsupervised Learning Phase*, each “petri dish” was subjected to a *Screening Phase* to determine which model instantiations produced image representations that are well-suited for performing invariant object recognition tasks.

During the *Screening Phase*, individual static images were supplied as input to each model, and the vector of responses from the units of its final layer were taken as that model's “representation” of the image. The labeled, “re-represented” images were then reduced in dimensionality by PCA and taken as inputs (training examples) for a classifier (in our case, a linear SVM).

**Figure 5:**
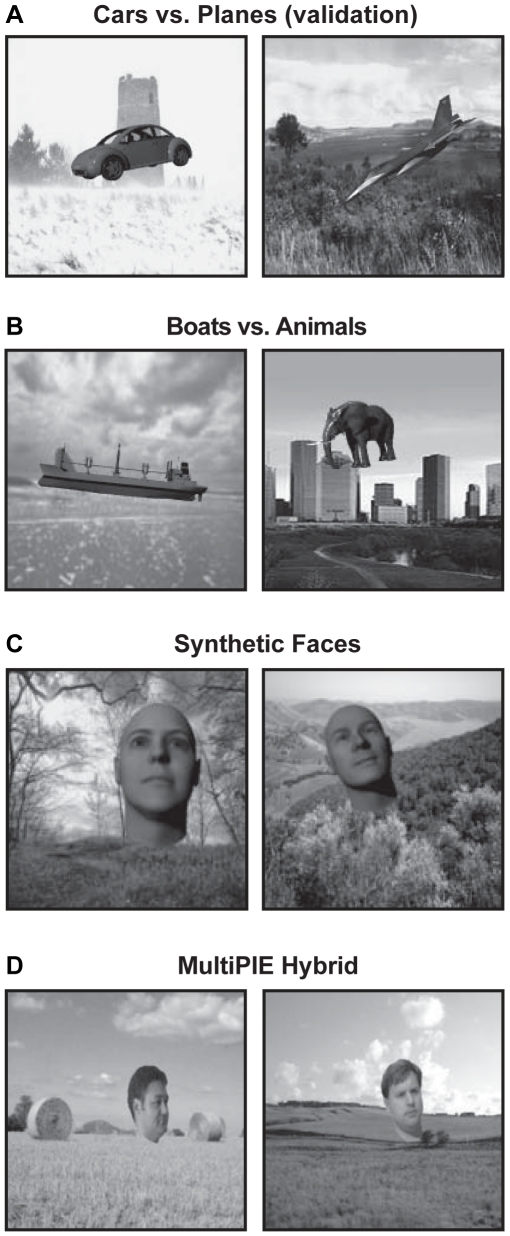
Examples of images from the validation test sets. (A) A new set of rendered cars and planes composited onto random natural backgrounds. (B) Rendered boats and animals. (C) Rendered female and male faces. (D) A subset of the MultiPIE face test set [27] with the faces manually removed from the background, and composited onto random image backgrounds, with additional variation in position, scale, and planar rotation added.

We used a simple “Cars vs. Planes” synthetic object recognition test as a screening task (see [11] for details). In this task, 3D models from two categories (cars and planes), were rendered across a wide range of variation in position, scale, view, and background. The rendered grayscale images (200 by 200 pixels) were provided as input to each model, and a classifier was trained to distinguish car images from plane images (150 training images per category). Performance of each model was then tested on a new set of unlabeled re-represented car and plane images (150 testing images per category). This recognition test has the benefit of being relatively quick to evaluate (because it only contains two classes), while at the same time having previous empirical grounding as a challenging object recognition test due to the large amount of position, scale, view, and background variation [11] (see Figure 5A).

#### Phase 4: validation

The best models selected during the *Screening Phase* were submitted to validation tests using other image sets, to determine if the representations generated by the models were useful beyond the immediate screening task. For the present work, four validation sets were used: 1) a new set of rendered cars and planes (generated by the same random process that generated the screening set, but with different specific examplars), 2) a set of rendered boats and animals 3) a set of rendered images of two synthetic faces (one male, one female, [10],[12]), and 4) a modified subset of the standard MultiPIE face recognition test set ([27]; here dubbed the “MultiPIE Hybrid” set). In the case of the rendered sets (sets 1–3), as with the screening set, the objects were rendered across a wide range of views, positions, and scales.

For the “MultiPIE hybrid” set, 50 images each of two individuals from the standard MultiPIE set were randomly selected from the full range of camera angles, lighting, expressions, and sessions included in the MultiPIE set. These faces were manually removed from their backgrounds and were further transformed in scale, position, planar rotation and were composited onto random natural backgrounds. Examples of the resulting images are shown in Figure 5.

For all sets (as with the screening set) classifiers were trained with labeled examples to perform a two-choice task (i.e. Cars vs. Planes, Boats vs. Animals, Face 1 vs. Face 2), and were subsequently tested with images not included in the training set.

While a number of standardized “natural” object and face recognition test sets exist [28]–[34], we made a deliberate choice not to use these sets. Previous investigations [10]–[12],[35],[36] have raised concerns with many of these sets, calling into question whether they appropriately capture the problem of interest. As a result, we chose to focus here on image sets that include substantial image variation by design, be they synthetic (as in our rendered set) or natural (as in the MultiPIE Hybrid set) in origin.

### Performance Comparison with Other Algorithms

#### “V1-like” baseline

Since object recognition performance measures are impossible to interpret in a vacuum, we used a simple *V1-like* model to serve as one baseline against which model performance can be compared. This *V1-like* model was taken, without modification, from Pinto et al. [11], and was shown previously to match or exceed the performance of a variety of purpose-built vision systems on the popular (but, we argue, flawed as a test of invariant object recognition) Caltech101 object recognition set and a wide variety of standard face recognition sets (ORL, Yale, CVL, AR, and Labeled Faces in the Wild [10],[12]). Importantly, this model is based on only a first-order description of the first stage of visual processing in the brain, and it contains no mechanisms that should allow it to tolerate the substantial image variation that makes object recognition hard in the first place [11],[19]. Here, this model serves as a lower bound on the amount of trivial regularity that exists in the test set. To be considered promising object recognition systems, models should at least exceed the performance of the *V1-like* model.

#### Comparison with state-of-the-art algorithms

To facilitate comparison with other models in the literature, we obtained code for, or re-implemented five “state of the art” object recognition algorithms from the extant literature: “Pyramid Histogram of Oriented Gradients” (PHOG) [37], “Pyramid Histogram of Words” (PHOW) (also known as the Spatial Pyramid [38]), the “Geometric Blur” shape descriptors [39], the descriptors from the “Scale Invariant Feature Transformation” (SIFT) [40], and the “Sparse Localized Features” (SLF) features of Mutch and Lowe [8] (a sparse extension of the C2 features from the Serre et al. HMAX model [7]). In all cases, we were able to reproduce or exceed the authors' reported performance for each system on the Caltech101 test set, which served as a sanity check that we had correctly implemented and used each algorithm as intended by its creators.

Each algorithm was applied using an identical testing protocol to our validation sets. In cases where an algorithm from the literature dictated that filters be optimized relative to each training set (e.g. [38] and [8]), we remained faithful to the authors' published descriptions and allowed this optimization, resulting in a different individually tailored model for each validation set. This was done even though our own high-throughput-derived models were not allowed such per-set optimizations (i.e. the same representation was used for all validation sets), and could therefore theoretically be “handicapped” relative to the state-of-the-art models.

## Results

### Object Recognition Performance

**Figure 6:**
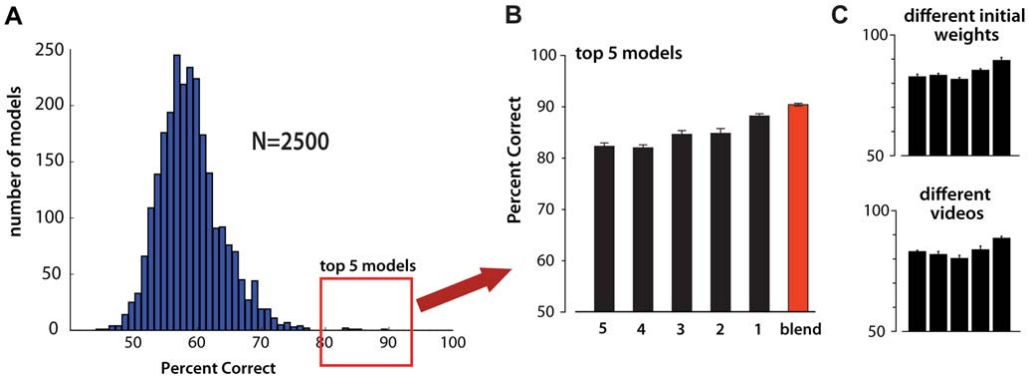
High-throughput screening in the “Law and Order” petri dish. (A) Histogram of the performance of 2,500 models on the “Cars vs. Planes” screening task (averaged over 10 random splits; error bars represent standard error of the mean). The top five performing models were selected for further analysis. (B) Performance of the top five models (1–5), and the performance achieved by averaging the five SVM kernels (red bar labelled “blend”) (C) Performance of the top five models (1–5) when trained with a different random initialization of filter weights (top) or with a different set of video clips taken from the “Law and Order” television program (bottom).

**Figure 7:**
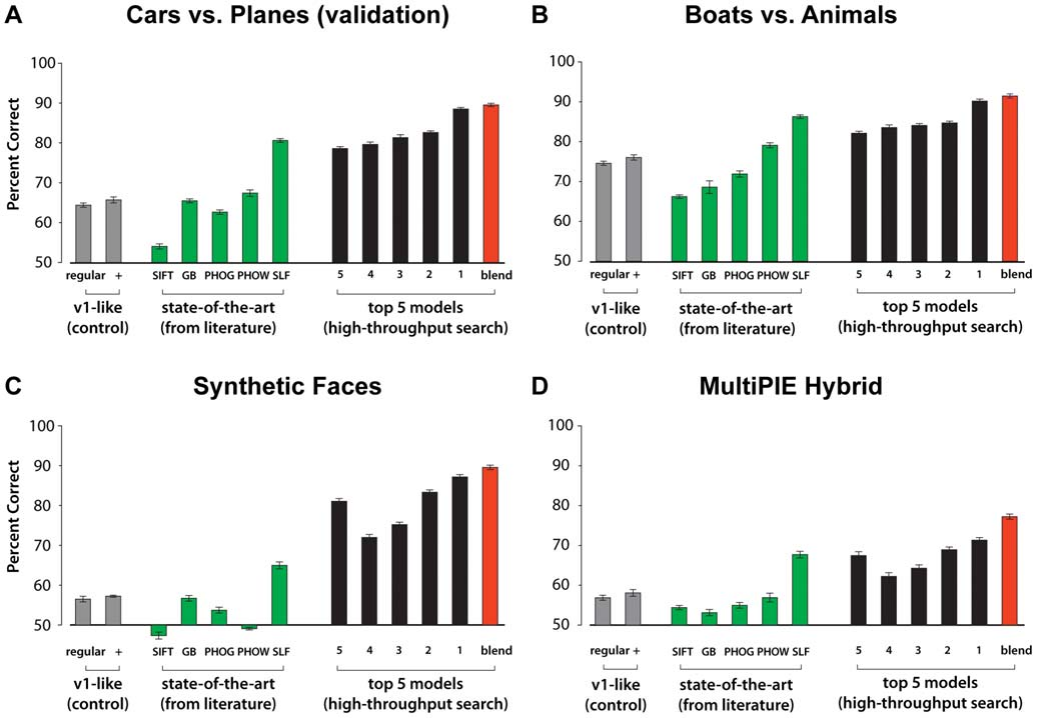
Validation. Performance of the top five models from the *Screening Phase* on a variety of other object recognition challenges. Example images from each object recognition test are shown in Figure 5. For each validation set, the performance (averaged over 10 random splits; error bars represent standard error of the mean) is first plotted for *V1-like* and *V1-like+* baseline models (see [10]–[12] for a detailed description of these two variants) (gray bars), and for five state-of-the-art vision systems (green bars): Scale Invariant Feature Transform (SIFT, [40]), Geometric Blur Descriptor (GB, [39]), Pyramidal Histogram of Gradients (PHOG, [37]), Pyramidal Histogram of Words (PHOW, [38]), and a biologically-inspired hierarchical model (“Sparse Localized Features” SLF, [8]). Finally, performance of the five best models derived from the high-throughput screening approach presented in this paper (black bars), and the performance achieved by averaging the five SVM kernels (red bar labelled “blend”). In general, high-throughput-derived models outperformed the *V1-like* baseline models, and tended to outperform a variety of state-of-the-art systems from the literature. Model instantiation 3281 and the blend of all five top models uniformly produced the best results across all test sets considered here.

As a first exploration of our high-throughput approach, we generated 7,500 model instantiations, in three groups of 2,500, with each group corresponding to a different class of unsupervised learning videos (“petri dishes”; see *Methods*). During the *Screening Phase*, we used the “Cars vs. Planes” object discrimination task [11] to assess the performance of each model, and the most promising five models from each set of 2,500 models was submitted to further analysis. The raw computation required to generate, train and screen these 7,500 models was completed in approximately one week, using 23 PlayStation 3 systems [41]. Results for models trained with the “Law and Order” petri dish during the *Unsupervised Learning Phase* are shown in Figure 6A. As expected, the population of randomly-generated models exhibited a broad distribution of performance on the screening task, ranging from chance performance (50%) to better than 80% correct. Figure 6B shows the performance of the best five models drawn from the pool of 2,500 models in the “Law and Order” petri dish. These models consistently outperformed the *V1-like* model baseline (Figure 7), and this performance was roughly maintained even when the model was retrained with a different video set (e.g. a different clip from Law and Order), or with a different random initialization of the filter kernel weights (Figure 6C).

Since these top models were selected for their high performance on the screening task, it is perhaps not surprising that they all show a high level of performance on that task. To determine whether the performance of these models generalized to other test sets, a series of *Validation* tests were performed. Specifically, we tested the best five models from each Unsupervised Learning petri dish on four test sets: two rendered object sets, one rendered face set, and a modified subset of the MultiPIE face recognition image set (see *Validation Phase* in Methods). Performance across each of these validation sets is shown in Figure 7 (black bars). While the exact ordering of model performance varied somewhat from validation set to validation set, the models selected during the *Screening Phase* performed well across the range of validation tasks.

The top five models found by our high-throughput screening procedure generally outperformed state-of-the-art models from the literature (see Methods) across all sets, with the best model found by the high-throughput search uniformly yielding the highest performance across all validation sets. Even greater performance was achieved by a simple summing of the SVM kernels from the top five models (red bar, Figure 7). Of note, the nearest contender from the set of state-of-the-art models is another biologically-inspired model [7],[8].

Interestingly, a large performance advantage between our high-throughput-derived models and state-of-the-art models was observed for the MultiPIE hybrid set, even though this is arguably the most different from the task used for screening, since it is composed from natural images (photographs), rather than synthetic (rendered) ones. It should be noted that several of the state-of-the-art models, including the sparse C2 features (“SLF” in Figure 7), which was consistently the nearest competitor to our models, used filters that were individually tailored to each validation test–i.e. the representation used for “Boats vs. Planes” was optimized for that set, and was different from the representation used for the MultiPIE Hybrid set. This is in contrast to our models, which learned their filters from a completely unrelated video data set (Law and Order) and were screened using an unrelated task (“Cars vs. Planes”, see Methods). While even better performance could no doubt be obtained by screening with a subset taken from each individual validation test, the generalizability of performance across a range of different tasks argues that our approach may be uncovering features and representations that are broadly useful. Such generality is in keeping with the models' biological inspiration, since biological visual representations must be flexible enough to represent a massive diversity of objects in order to be useful.

Results for the 2,500 models in each of the other two “petri dishes” (i.e. models trained with alternate video sets during unsupervised learning) were appreciably similar, and are shown in Supplemental Figures S7 and S8, using the same display conventions set forth in Figures 6 and 7.

## Discussion

We have demonstrated a high-throughput framework, within which a massive number of candidate vision models can be generated, screened, and analyzed. Models found in this way were found to consistently outperform an experimentally-motivated baseline model (a *V1-like* model; [10]–[12]), and the representations of visual space instantiated by these models were found to be useful generally across a variety of object recognition tasks. The best of these models and the blend of the five best models were both found to consistently outperform a variety of state-of-the-art machine vision systems for all of the test sets explored here, even without any additional optimization.

This work builds on a long tradition of machine vision systems inspired by biology (e.g. [1]–[4],[7],[9]). However, while this past work has generated impressive progress towards building artificial visual systems, it has explored only a few examples drawn from the larger space of biologically-inspired models. While the task of exploring the full space of possible model instantiations remains daunting (even within the relatively restricted “first-order” class of models explored here), our results suggest that even a relatively simple, brute-force high-throughput search strategy is effective in identifying promising models for further study. In the parameter space used here, we found that a handful of model instantiations performed substantially better than the rest, with these “good” models occurring at a rate of approximately one in five-hundred. The relative rarity of these models underscores the importance of performing large-scale experiments with many model instantiations, since these models would be easy to miss in a “one-off” mode of exploration. Importantly, these rare, high-performing models performed well across a range of object recognition tasks, indicating that our approach does not simply optimize for a given task, but can uncover visual representations of general utility.

Though not conceptually critical to our approach, modern graphics hardware played an essential role in making our experiments possible. In approximately one week, we were able to test 7,500 model instantiations, which would have taken approximately two years using a conventional (e.g. MATLAB-based) approach. While it is certainly possible to use better-optimized CPU-based implementations, GPU hardware provides large increases in attainable computational power (see Figure 1 and Supplemental Figure S1).

An important theme in this work is the use of parametrically controlled objects as a way of guiding progress. While we are ultimately interested in building systems that tolerate image variation in real-world settings, such sets are difficult to create, and many popular currently-available “natural” object sets have been shown to lack realistic amounts of variation [10]–[12]. Our results show that it is possible to design a small synthetic set to screen and select models that generalize well across various visual classification tasks, suggesting that parametric sets can capture the essence of the invariant object recognition problem. Another critical advantage of the parametric screening approach presented here is that task difficulty can be increased on demand–that is, as models are found that succeed for a given level of image variation, the level of variation (and therefore the level of task difficulty), can be “ratcheted up” as well, maintaining evolutionary “pressure” towards better and better models.

While we have used a variety of synthetic (rendered) object image sets, images need not be synthetic to meet the requirements of our approach. The modified subset of the MultiPIE set used here (“MultiPIE Hybrid”, Figure 5) is an example of how parametric variation can also be achieved using carefully controlled photography.

### Future Directions

While our approach has yielded a first crop of promising biologically-inspired visual representations, it is another, larger task to understand how these models work, and why they are better than other alternatives. While such insights are beyond the scope of the present paper, our framework provides a number of promising avenues for further understanding.

**Figure 8:**
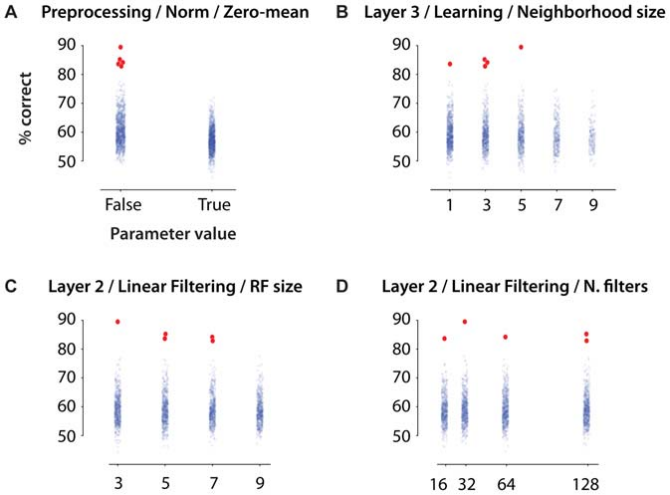
Distributions of screening task performance, as a function of parameter values for four arbitrarily-chosen parameters. See Supplemental Text S1 for an exhaustive description of the meaning of each parameter. The top five best performing models are plotted in red, with the other models overplotted in semi-transparent blue. The parameters considered in (A) and (B) show hints of a relationship between parameter value and inclusion in the top five. In (A) all of the five best models had the same value of the parameter, and in (B) best models were clustered in lower ranges of parameter value. (C) and (D) show parameters where the best models were distributed across a range of parameter values. Such examinations of parameter values are in no way conclusive, but can provide hints as to which parameters might be important for performance. See also Supplemental Text S3, Figures S9 and S10.

One obvious direction is to directly analyze the parameter values of the best models in order to understand which parameters are critical for performance. Figure 8 shows distributions of parameter values for four arbitrarily chosen parameters. While in no way conclusive, there are hints that some particular parameter values may be more important for performance than others (for quantitative analysis of the relationship between model parameters and performance, see Supplemental Text S3, Figures S9 and S10). The speed with which large collections of models can be evaluated opens up the possibility of running large-scale experiments where given parameters are held fixed, or varied systematically. Insights derived from such experiments can then be fed back into the next round of high-throughput search, either by adjusting the parameter search space or by fundamentally adjusting the algorithm itself. Such iterative refinement is an active area of research in our group.

The search procedure presented here has already uncovered promising visual representations, however, it represents just the simplest first step one might take in conducting a large-scale search. For the sake of minimizing conceptual complexity, and maximizing the diversity of models analyzed, we chose to use random, brute-force search strategy. However, a rich set of search algorithms exist for potentially increasingly the efficiency with which this search is done (e.g. genetic algorithms [42], simulated annealing [43], and particle swarm techniques [44]). Interestingly, our brute-force search found strong models with relatively high probability, suggesting that, while these models would be hard to find by “manual” trial-and-error, they are not especially rare in the context of our high-throughput search.

While better search algorithms will no doubt find better instances from the model class used here, an important future direction is to refine the parameter-ranges searched and to refine the algorithms themselves. While the model class described here is large, the class of all models that would count as “biologically-inspired” is even larger. A critical component of future work will be to adjust existing mechanisms to achieve better performance, and to add new mechanisms (including more complex features such as long-range feedback projections). Importantly, the high-throughput search framework presented here provides a coherent means to find and compare models and algorithms, without being unduly led astray by weak sampling of the potential parameter space.

Another area of future work is the application of high-throughput screening to new problem domains. While we have here searched for visual representations that are good for object recognition, our approach could also be applied to a variety of other related problems, such as object tracking, texture recognition, gesture recognition, feature-based stereo-matching, etc. Indeed, to the extent that natural visual representations are flexibly able to solve all of these tasks, we might likewise hope to mine artificial representations that are useful in a wide range of tasks.

Finally, as the scale of available computational resources steadily increases, our approach naturally scales as well, allowing more numerous, larger, and more complex models to be examined. This will give us both the ability to generate more powerful machine vision systems, and to generate models that better match the scale of natural systems, providing more direct footing for comparison and hypothesis generation. Such scaling holds great potential to accelerate both artificial vision research, as well as our understanding of the computational underpinnings of biological vision.

## Supporting Information

Figure S1 Processing Performance of the Linear Filtering Operation. The theoretical and observed processing performance in GFLOPS (billions of floating point operations per second) is plotted for a key filtering operation in our biologically-inspired model implementation. Theoretical performance numbers were taken from manufacturer marketing materials and are generally not achievable in real-world conditions, as they consider multiple floating operations per clock cycle, without regard to memory communication latencies (which typically are the key determinant of real-world performance). Observed processing performance for the filtering operation varied across candidate models in the search space, as input and filter sizes varied. Note that the choice of search space can be adjusted to take maximum advantage of the underlying hardware at hand. We plot the “max” observed performance for a range of CPU and GPU implementations, as well as the “mean” and “min” performance of our PlayStation 3 implementation observed while running the 7,500 models presented in this study. The relative speedup denotes the peak performance ratio of our optimized implementations over a reference MATLAB code on one of the Intel QX9450's core (e.g. using filter2, which is itself coded in C++), whereas the relative GFLOPS per dollar indicates the peak performance per dollar ratio. Costs of typical hardware for each approach and cost per FLOPS are shown at the bottom. * These ranges indicate the performance and cost of a single system containing from one (left) to four (right) GPUs. ** These costs include both the hardware and MATLAB yearly licenses (based on an academic discount pricing, for one year).
(1.19 MB TIF)

Figure S2 A schematic of the flow of transformations performed in our family of biologically-inspired models. Blue-labeled boxes indicate the cascade of operations performed in each of the three layers in the canonical model. Gray-labeled boxes to the right indicate filter weight update steps that take place during the Unsupervised Learning Phase after the processing of each input video frame. The top gray-labeled box shows processing steps undertaken during the Screening and Validation Phases to evaluate the performance achievable with each model instantiation.
(0.95 MB TIF)

Figure S3 Examples of Layer 1 filters taken from different models. A random assortment of linear filter kernels taken from the first layers of the top five (A) and fifteen randomly chosen other model instantiations (B) taken from the “Law and Order” petri dish. Each square represents a single two-dimensional filter kernel, with the values of each filter element represented in gray scale (the gray-scale is assigned on a per-filter basis, such that black is the smallest value found in the kernel, and white is the largest). For purposes of comparison, a fixed number of filters were taken from each model's Layer 1, even though different models have differing number of filters in each layer. Filter kernels are initialized with random values and learn their structure during the Unsupervised Learning Phase of model generation. Interestingly, oriented structures are common in filter from both the top five models and from non-top-five models.
(3.71 MB TIF)

Figure S4 Examples of Layer 2 filters taken from different models. Following the same basic convention as in Supplemental Figure S3, a random assortment of portions of filter kernels from Layer 2 of the top five (A) and fifteen other randomly-chosen model instantiations (B) are shown in gray-scale to provide a qualitative sense of what the linear filters (produced as a result of the Unsupervised Learning Phase) look like. Note that since each Layer 1 is itself a stack of k^l = 1^ two-dimensional planes (or “feature maps”) resulting from filtering with a stack of k^l = 1^ filters (see Supplemental Text S1 and Supplemental Figure S6, each Layer 2 filter is actually a f_s_^l = 2^ × f_s_^l = 2^ × k^l = 1^ kernel For the sake of visual clarity, we here present just one randomly-chosen f_s_^l = 2^ × f_s_^l = 2^ “slice” from each of the randomly-chosen filters. As in Supplemental Figure S3, there are signs of “structure” in the filters of both the top five and non-top-five models.
(3.76 MB TIF)

Figure S5 Examples of Layer 3 filters taken from different models. Following the same basic convention as in Supplemental Figures S3 and S4, a random assortment of portions of filter kernels from Layer 3 of the top five (A) and fifteen other randomly-chosen model instantiations (B) are shown in gray-scale to provide a qualitative sense of what the linear filters (produced as a result of the Unsupervised Learning Phase) look like. Note that since each Layer 2 is itself a stack of k^l = 2^ two-dimensional planes (or “feature maps”) resulting from filtering with a stack of k^l = 2^ filters (see Supplemental Text S1 and Supplemental Figure S6), each Layer 3 filter is actually a f_s_^l = 3^ × f_s_^l = 3^ × k^l = 2^ kernel. For the sake of visual clarity, we here present just one randomly-chosen f_s_^l = 3^ × f_s_^l = 3^ “slice” from each of the randomly-chosen filters. As in Supplemental Figures S3 and S4, there are signs of “structure” in the filters of both the top five and non-top-five models.
(3.72 MB TIF)

Figure S6 Example filterbanks from the best model instantiation in the “Law and Order” Petri Dish. Filter kernels were learned during the Unsupervised Learning Phase, after which filter weights were fixed. Colors indicate filter weights, and were individually normalized to make filter structure clearer (black-body color scale with black indicating the smallest filter weight, white representing the largest filter weight). The filter stack for each layer consists of k^l^ filters, with size f_s_. Because the Layer 1 filterbank for this model includes 16 filters, the Layer 1 output will have a feature “depth” of 16, and thus each Layer 2 filter is a stack of 16 f_s_ × f_s_ kernels. One filter (filter 61) is shown expanded for illustration purposes. Similarly, since the Layer 2 filterbank in this example model includes 64 filters, the output of Layer 2 will have a depth of 64, and thus each filter in Layer 3 filterbank must also be 64-deep.
(1.65 MB TIF)

Figure S7 High-throughput screening in the “Cars and Planes” Petri Dish. Data are shown according to the same display convention set forth in the main paper. (A) Histogram of the performance of 2,500 models on the “Cars vs. Planes” screening task. The top five performing models were selected for further analysis. (B) Performance of the top five models (1–5). (C) Performance of the top five models when trained with a different random initialization of filter weights (top) or with a different set of video clips (bottom). (D) Performance of the top five models from the Screening Phase on a variety of other object recognition challenges.
(0.21 MB TIF)

Figure S8 High-throughput screening and validation in the “Boats'” Petri Dish. Data are shown according to the same display convention set forth in the main paper. (A) Histogram of the performance of 2,500 models on the “Cars vs. Planes” screening task. The top five performing models were selected for further analysis. (B) Performance of the top five models (1–5). (C) Performance of the top five models when trained with a different random initialization of filter weights (top) or with a different set of video clips (bottom). (D) Performance of the top five models from the Screening Phase on a variety of other object recognition challenges.
(0.22 MB TIF)

Figure S9 Linear regression analysis of relationship between parameter values and model performance. As a first-order analysis of the relationship between model parameters and model performance, we performed a linear regression analysis in which the values of each of the 52 parameters were included as predictors in a multiple linear regression analysis. Next, p-values were computed for the t statistic on each beta weight in the regression. A histogram of the negative natural log of the p-values is shown here, with the bin including significant p-values highlighted in orange (each count corresponds to one model parameter). For reference, the histogram is divided into three ranges (low-nonsignificant, medium-nonsignificant, and significant) and a listing of parameters included each significance range is printed below the histogram. Each parameter listing includes a 1) verbal description of the parameter, 2) its symbol according to the terminology in the Supplemental Methods, 3) the section number where it is referenced, and 4) whether it was positively (“+”) or negatively (“−”) correlated with performance. In addition, the parameters were divided into three rough conceptual groups and were color-coded accordingly: Filtering (green), Normalization/Activation/Pooling (red), and Learning (blue). Beneath the bin corresponding to significantly predictive parameters, a bar plot showing the fraction of each group found in the set of significant parameters. The expected fraction, if the parameters were distributed randomly, is shown as a dotted line. Activation/Normalization/Pooling parameters were slightly over-represented in the set of significantly-predictive parameters, but no group was found to be significantly over- or under-represented (p = 0.338; Fischer's exact test).
(2.28 MB TIF)

Figure S10 How similar are the top models? (A) Model similarity on the basis of parameter values (L0 or Hamming Distance). Each model is specified by a vector of 52 parameter values. As a first attempt at comparing models, we generated an expanded binary parameter vector in which every possible parameter/value combination was represented as a separate variable (e.g. a parameter ω that can take on values 3, 5, and 7 would be included in the expanded vector as three binary values [ω = 3], [ω = 5], and [ω = 7]). The Hamming distance distance between any two vectors can then serve as a metric of the similarity between any two models. In order to determine if the top five models taken from the “Law and Order” petri dish were more similar to each than would be expected of five randomly selected models, we computed the median pairwise Hamming distance between the top five models, and between a random sampling of 100,000 sets of five models taken from the remaining (non-top-five) models. The distribution of randomly selected model pairs is shown in (A), and the observed median distance amongst the top five models is indicated by an arrow. The top-five models tended to be more similar to one another than to a random selection of models from the full population, but this effect was not significant (p = 0.136; permutation test). (B) Model similarity on the basis of output (“Representation” similarity). As another way to compare model similarity, for each model we computed model output vectors for a selection of 600 images taken from the Screening task image sets. We then computed the L2 (Euclidean) distance matrix between these “re-represented” image vectors as a proxy for the structure of the output space of each model. A distance metric between any two models was then defined as the L2 distance between the unrolled upper-diagonal portion of the two models' similarity matrices (this distance metric is similar to the Frobenius norm). Finally, as in (A), the median distances between the top five models and between a collection of 10,000 randomly drawn sets of five models were computed. The histogram in (B) shows the distribution of median distances from randomly drawn sets of five models, and the arrow indicates the median distance observed in the top-five set. As in (A), the top-five models tended to be more similar to one another (lower distance), but this effect was not significant (p = 0.082; permutation test).
(6.31 MB TIF)

Text S1 Search Space of Candidate Models.
(0.14 MB PDF)

Text S2 Technical Details of the Computational Framework.
(0.08 MB PDF)

Text S3 First-Order Analyses of Model Parameters and Behavior.
(0.05 MB PDF)
