# Supplementary figures and images for: A High-Throughput Screening Approach to Discovering Good Forms of Biologically Inspired Visual Representation
Source: PLoS Comput Biol. 2009 Nov 26;5(11):e1000579. doi: 10.1371/journal.pcbi.1000579 (PMC2775908; doi:10.1371/journal.pcbi.1000579)

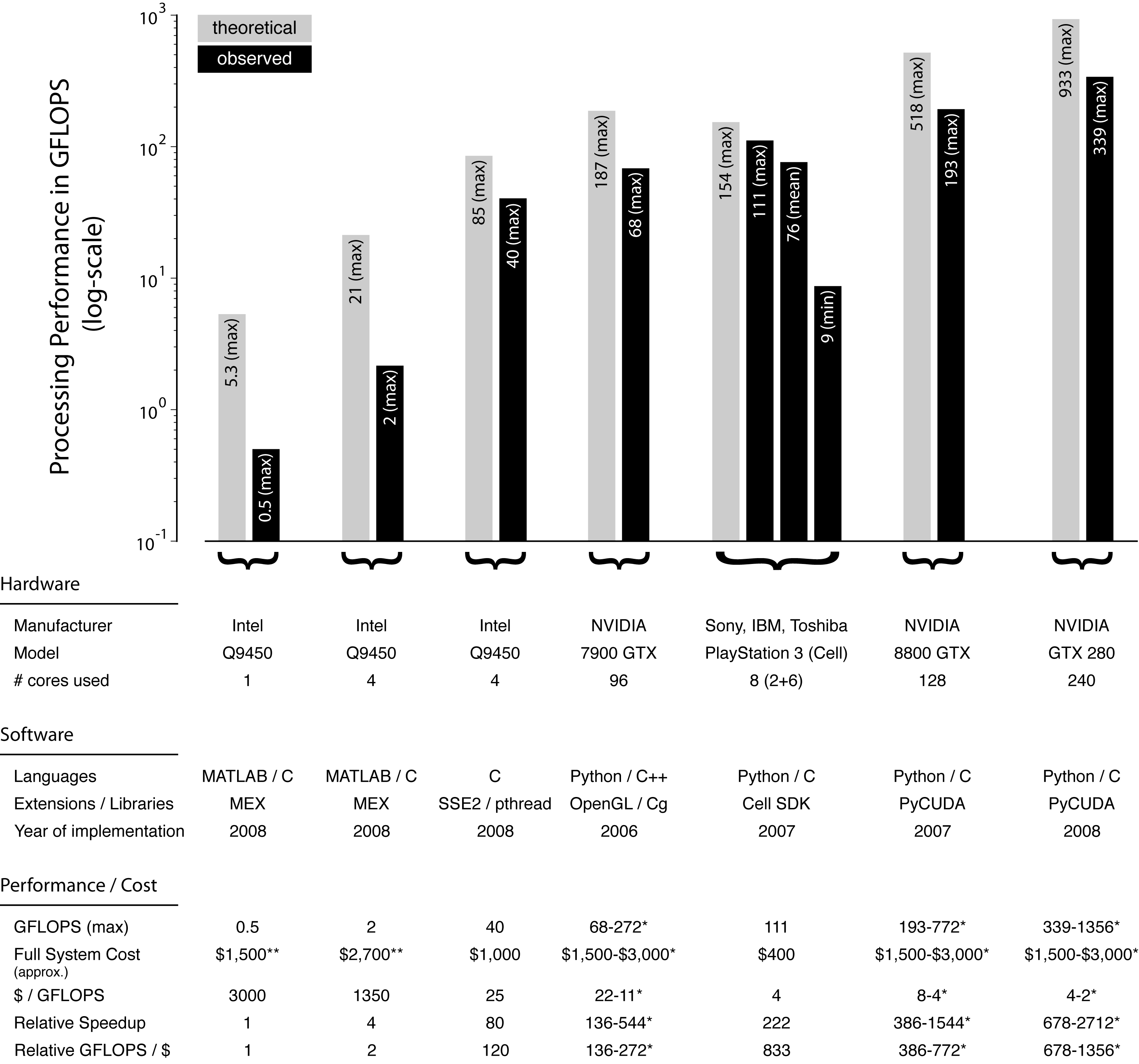

Supplement: Figure S1 — Processing Performance of the Linear Filtering Operation. The theoretical and observed processing performance in GFLOPS (billions of floating point operations per second) is plotted for a key filtering operation in our biologically-inspired model implementation. Theoretical performance numbers were taken from manufacturer marketing materials and are generally not achievable in real-world conditions, as they consider multiple floating operations per clock cycle, without regard to memory communication latencies (which typically are the key determinant of real-world performance). Observed processing performance for the filtering operation varied across candidate models in the search space, as input and filter sizes varied. Note that the choice of search space can be adjusted to take maximum advantage of the underlying hardware at hand. We plot the “max” observed performance for a range of CPU and GPU implementations, as well as the “mean” and “min” performance of our PlayStation 3 implementation observed while running the 7,500 models presented in this study. The relative speedup denotes the peak performance ratio of our optimized implementations over a reference MATLAB code on one of the Intel QX9450's core (e.g. using filter2, which is itself coded in C++), whereas the relative GFLOPS per dollar indicates the peak performance per dollar ratio. Costs of typical hardware for each approach and cost per FLOPS are shown at the bottom. * These ranges indicate the performance and cost of a single system containing from one (left) to four (right) GPUs. ** These costs include both the hardware and MATLAB yearly licenses (based on an academic discount pricing, for one year). (1.19 MB TIF) [file pcbi.1000579.s001.tif]

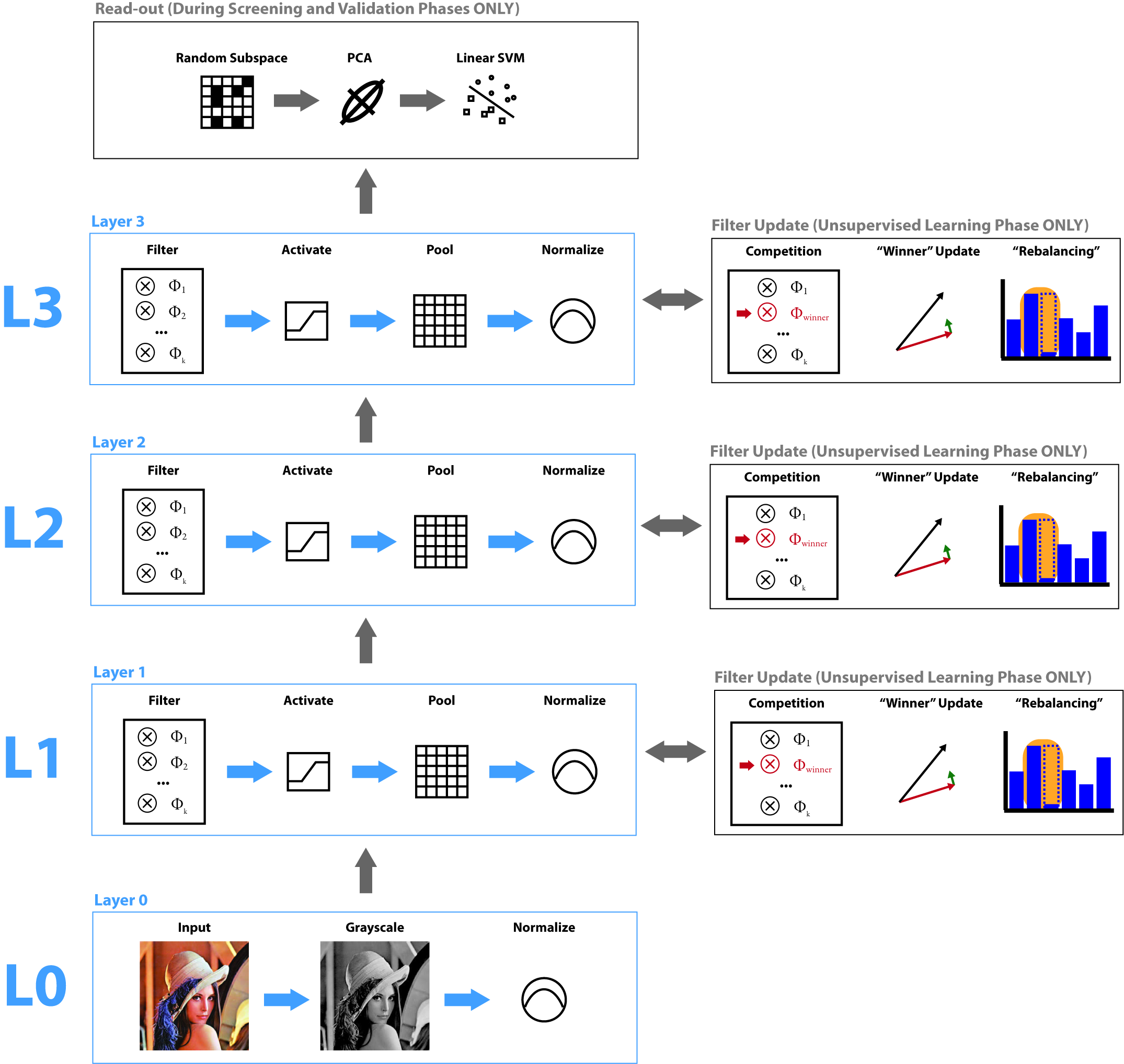

Supplement: Figure S2 — A schematic of the flow of transformations performed in our family of biologically-inspired models. Blue-labeled boxes indicate the cascade of operations performed in each of the three layers in the canonical model. Gray-labeled boxes to the right indicate filter weight update steps that take place during the Unsupervised Learning Phase after the processing of each input video frame. The top gray-labeled box shows processing steps undertaken during the Screening and Validation Phases to evaluate the performance achievable with each model instantiation. (0.95 MB TIF) [file pcbi.1000579.s002.tif]

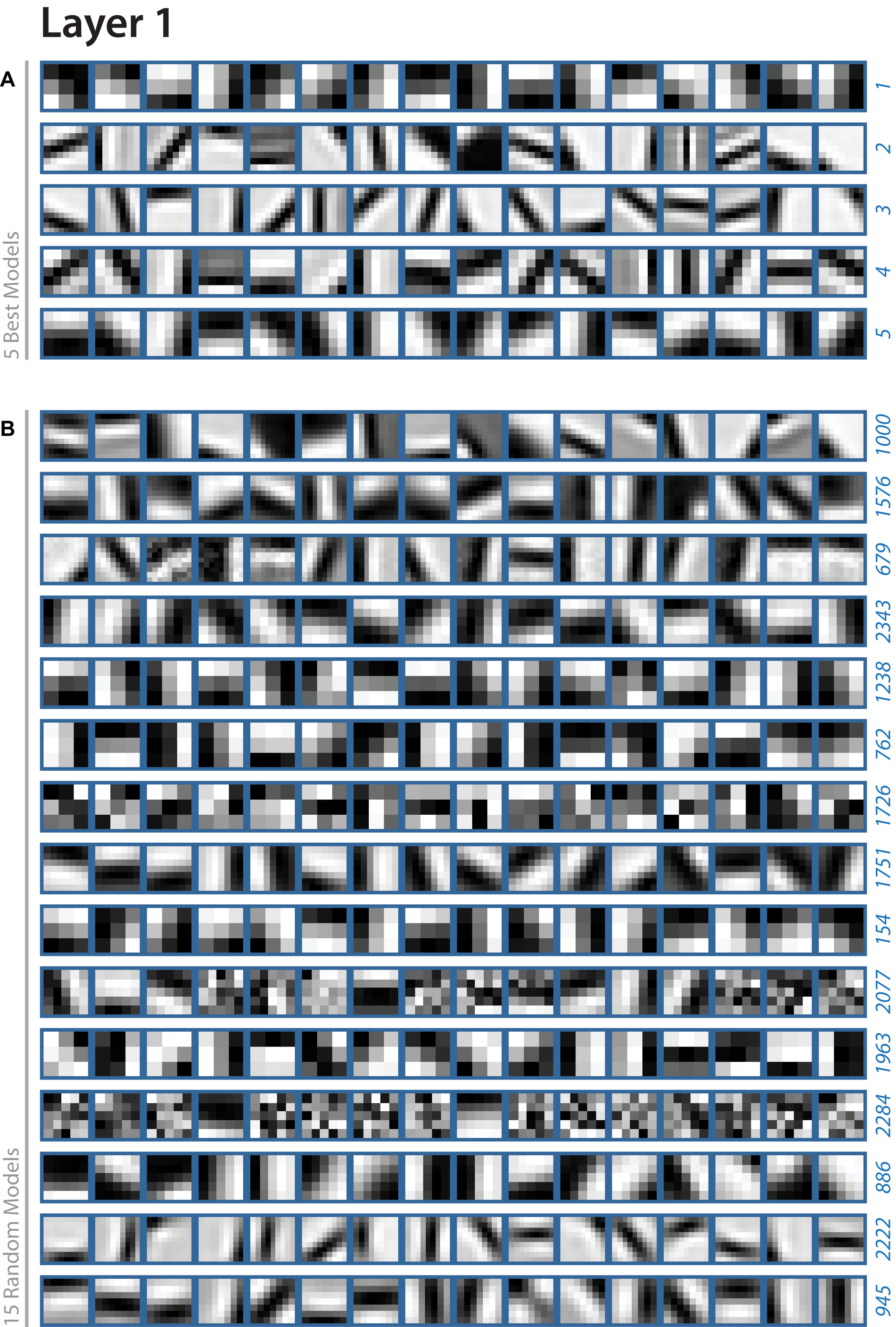

Supplement: Figure S3 — Examples of Layer 1 filters taken from different models. A random assortment of linear filter kernels taken from the first layers of the top five (A) and fifteen randomly chosen other model instantiations (B) taken from the “Law and Order” petri dish. Each square represents a single two-dimensional filter kernel, with the values of each filter element represented in gray scale (the gray-scale is assigned on a per-filter basis, such that black is the smallest value found in the kernel, and white is the largest). For purposes of comparison, a fixed number of filters were taken from each model's Layer 1, even though different models have differing number of filters in each layer. Filter kernels are initialized with random values and learn their structure during the Unsupervised Learning Phase of model generation. Interestingly, oriented structures are common in filter from both the top five models and from non-top-five models. (3.71 MB TIF) [file pcbi.1000579.s003.tif]

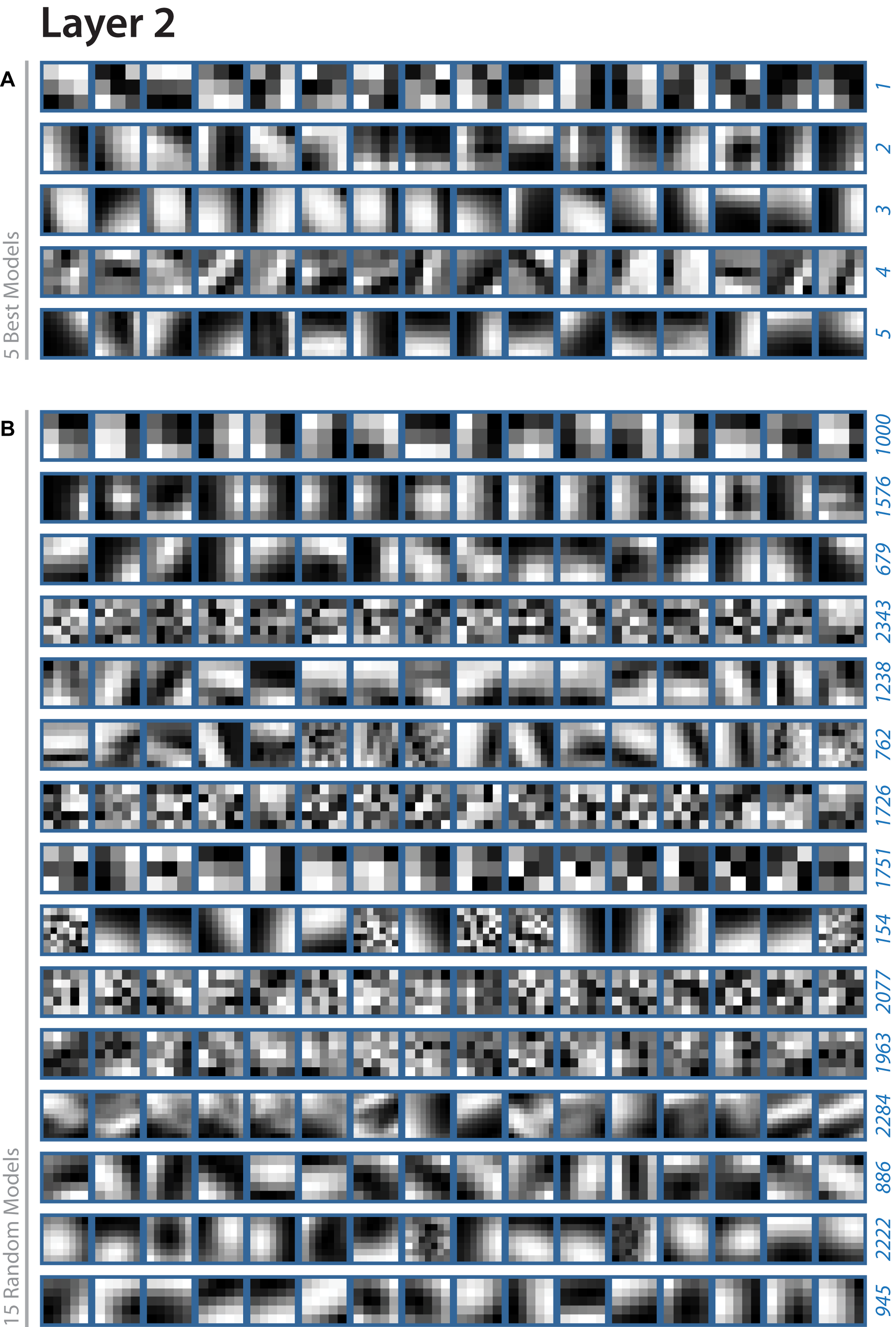

Supplement: Figure S4 — Examples of Layer 2 filters taken from different models. Following the same basic convention as in Supplemental Figure S3, a random assortment of portions of filter kernels from Layer 2 of the top five (A) and fifteen other randomly-chosen model instantiations (B) are shown in gray-scale to provide a qualitative sense of what the linear filters (produced as a result of the Unsupervised Learning Phase) look like. Note that since each Layer 1 is itself a stack of kl = 1 two-dimensional planes (or “feature maps”) resulting from filtering with a stack of kl = 1 filters (see Supplemental Text S1 and Supplemental Figure S6, each Layer 2 filter is actually a fs l = 2 × fs l = 2 × kl = 1 kernel For the sake of visual clarity, we here present just one randomly-chosen fs l = 2 × fs l = 2 “slice” from each of the randomly-chosen filters. As in Supplemental Figure S3, there are signs of “structure” in the filters of both the top five and non-top-five models. (3.76 MB TIF) [file pcbi.1000579.s004.tif]

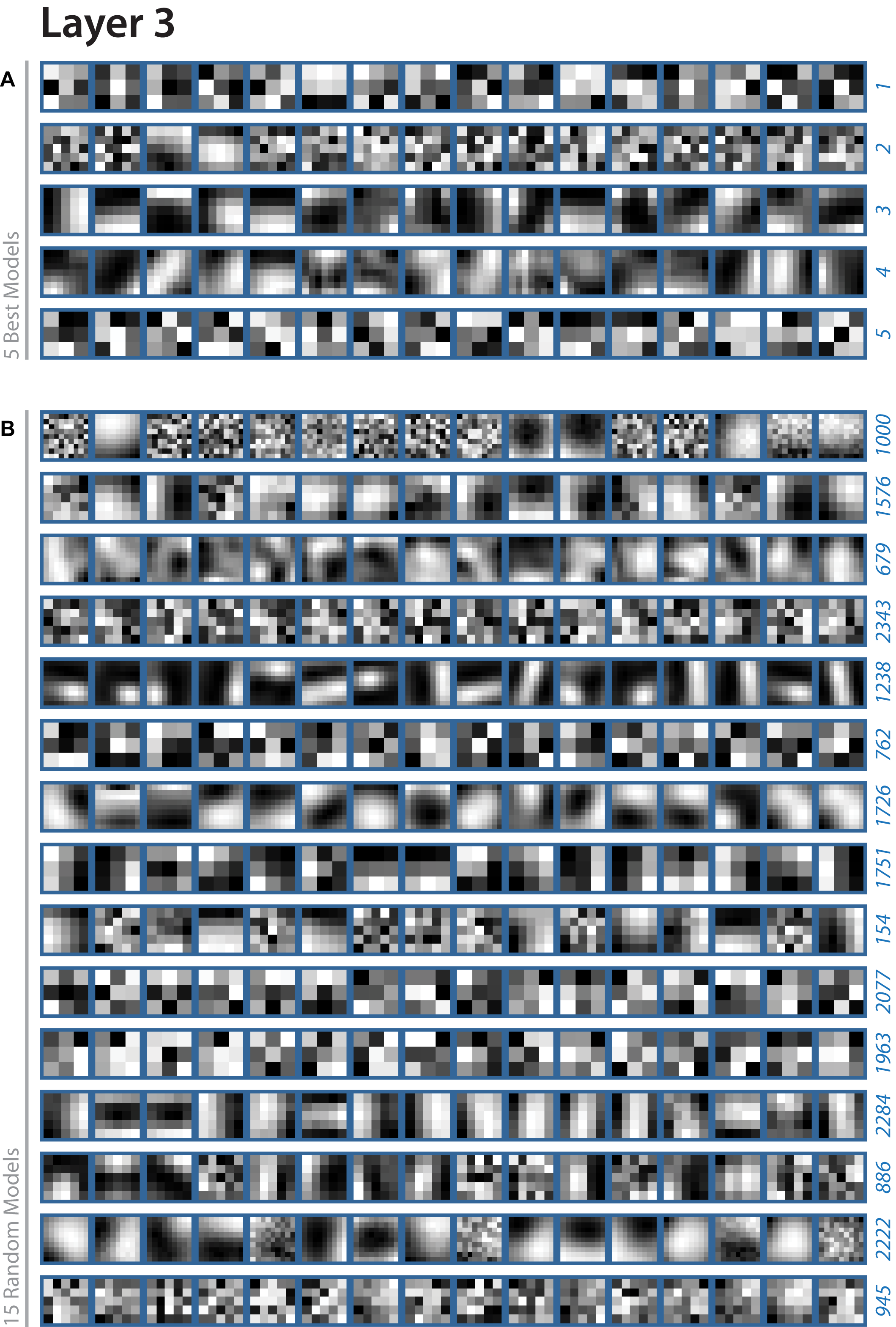

Supplement: Figure S5 — Examples of Layer 3 filters taken from different models. Following the same basic convention as in Supplemental Figures S3 and S4, a random assortment of portions of filter kernels from Layer 3 of the top five (A) and fifteen other randomly-chosen model instantiations (B) are shown in gray-scale to provide a qualitative sense of what the linear filters (produced as a result of the Unsupervised Learning Phase) look like. Note that since each Layer 2 is itself a stack of kl = 2 two-dimensional planes (or “feature maps”) resulting from filtering with a stack of kl = 2 filters (see Supplemental Text S1 and Supplemental Figure S6), each Layer 3 filter is actually a fs l = 3 × fs l = 3 × kl = 2 kernel. For the sake of visual clarity, we here present just one randomly-chosen fs l = 3 × fs l = 3 “slice” from each of the randomly-chosen filters. As in Supplemental Figures S3 and S4, there are signs of “structure” in the filters of both the top five and non-top-five models. (3.72 MB TIF) [file pcbi.1000579.s005.tif]

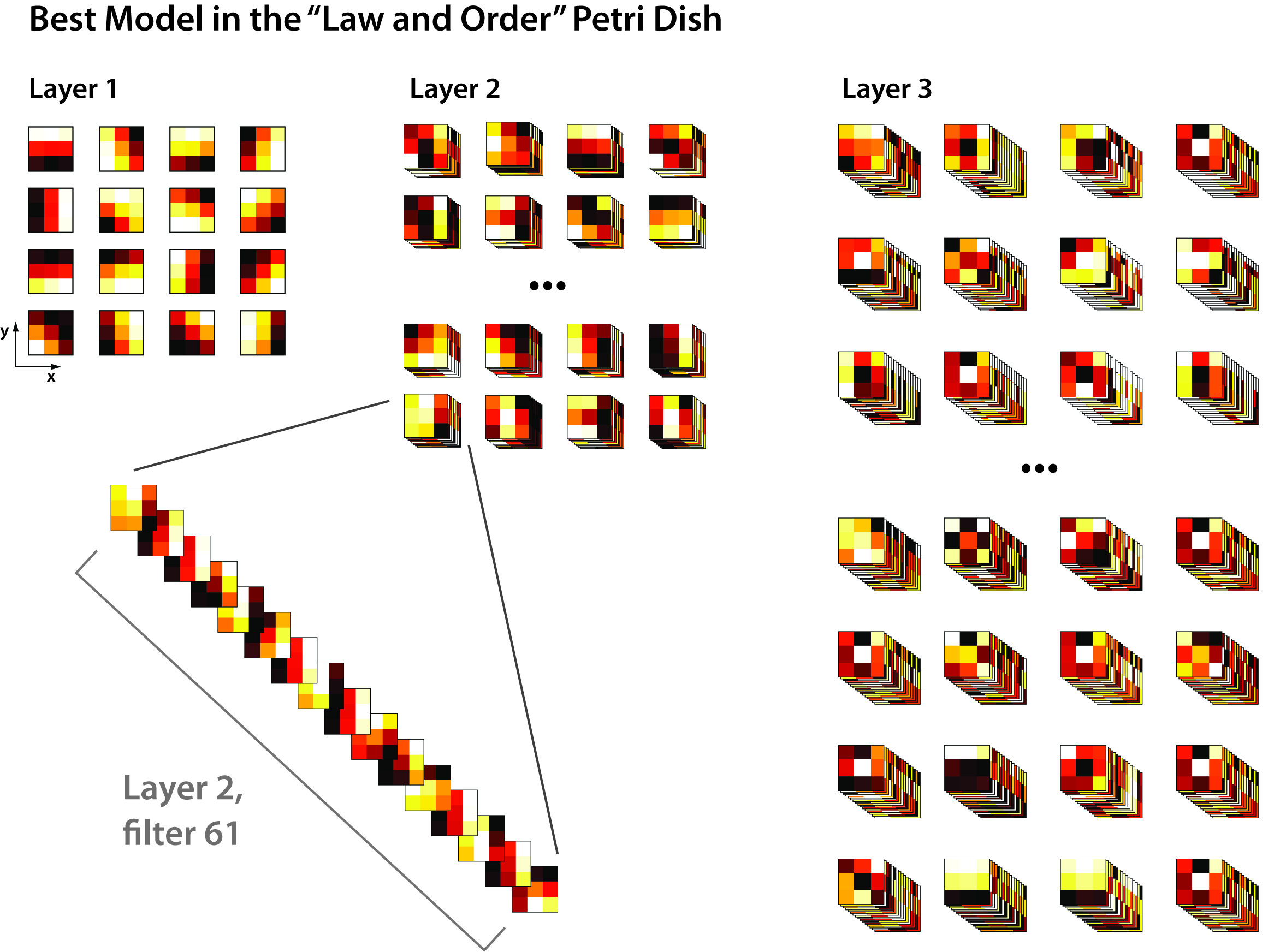

Supplement: Figure S6 — Example filterbanks from the best model instantiation in the “Law and Order” Petri Dish. Filter kernels were learned during the Unsupervised Learning Phase, after which filter weights were fixed. Colors indicate filter weights, and were individually normalized to make filter structure clearer (black-body color scale with black indicating the smallest filter weight, white representing the largest filter weight). The filter stack for each layer consists of kl filters, with size fs. Because the Layer 1 filterbank for this model includes 16 filters, the Layer 1 output will have a feature “depth” of 16, and thus each Layer 2 filter is a stack of 16 fs × fs kernels. One filter (filter 61) is shown expanded for illustration purposes. Similarly, since the Layer 2 filterbank in this example model includes 64 filters, the output of Layer 2 will have a depth of 64, and thus each filter in Layer 3 filterbank must also be 64-deep. (1.65 MB TIF) [file pcbi.1000579.s006.tif]

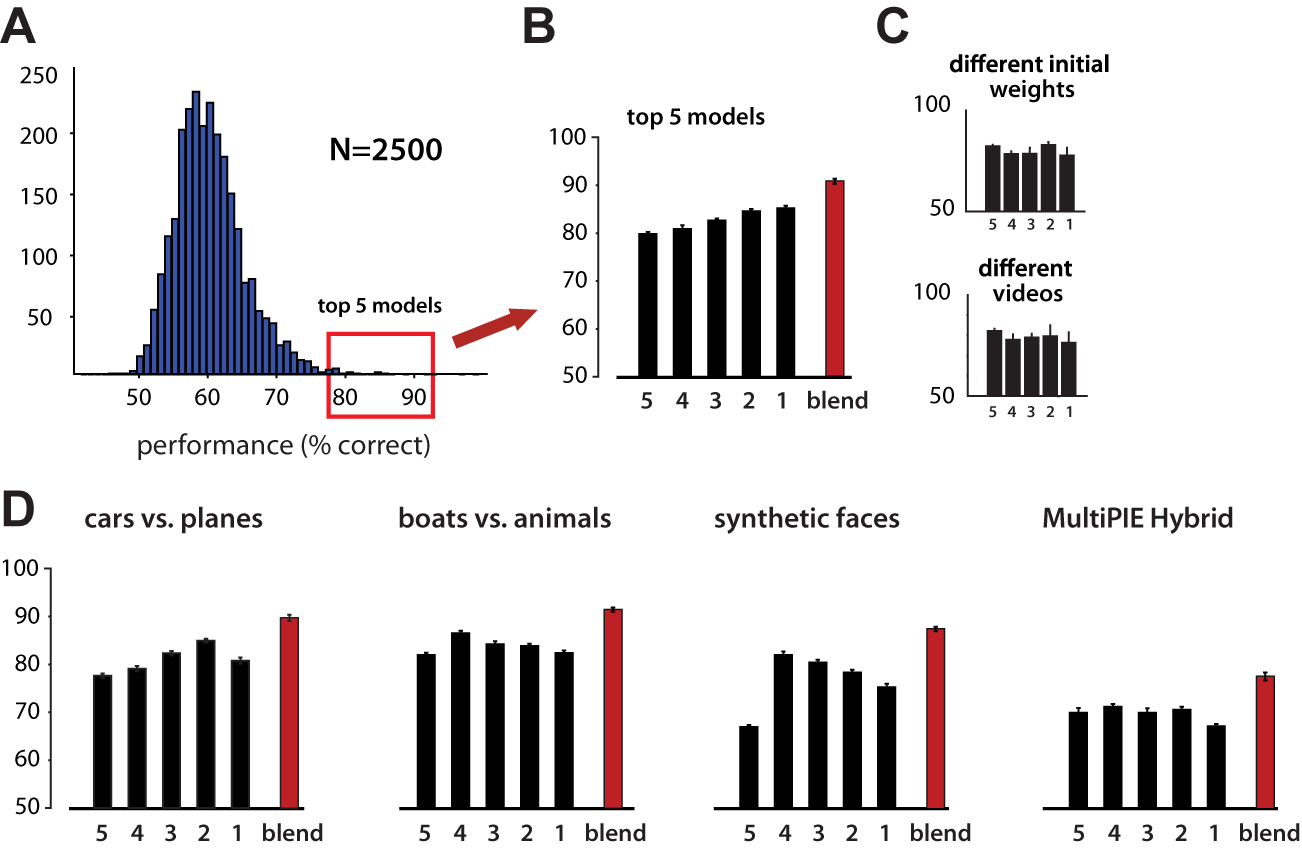

Supplement: Figure S7 — High-throughput screening in the “Cars and Planes” Petri Dish. Data are shown according to the same display convention set forth in the main paper. (A) Histogram of the performance of 2,500 models on the “Cars vs. Planes” screening task. The top five performing models were selected for further analysis. (B) Performance of the top five models (1–5). (C) Performance of the top five models when trained with a different random initialization of filter weights (top) or with a different set of video clips (bottom). (D) Performance of the top five models from the Screening Phase on a variety of other object recognition challenges. (0.21 MB TIF) [file pcbi.1000579.s007.tif]

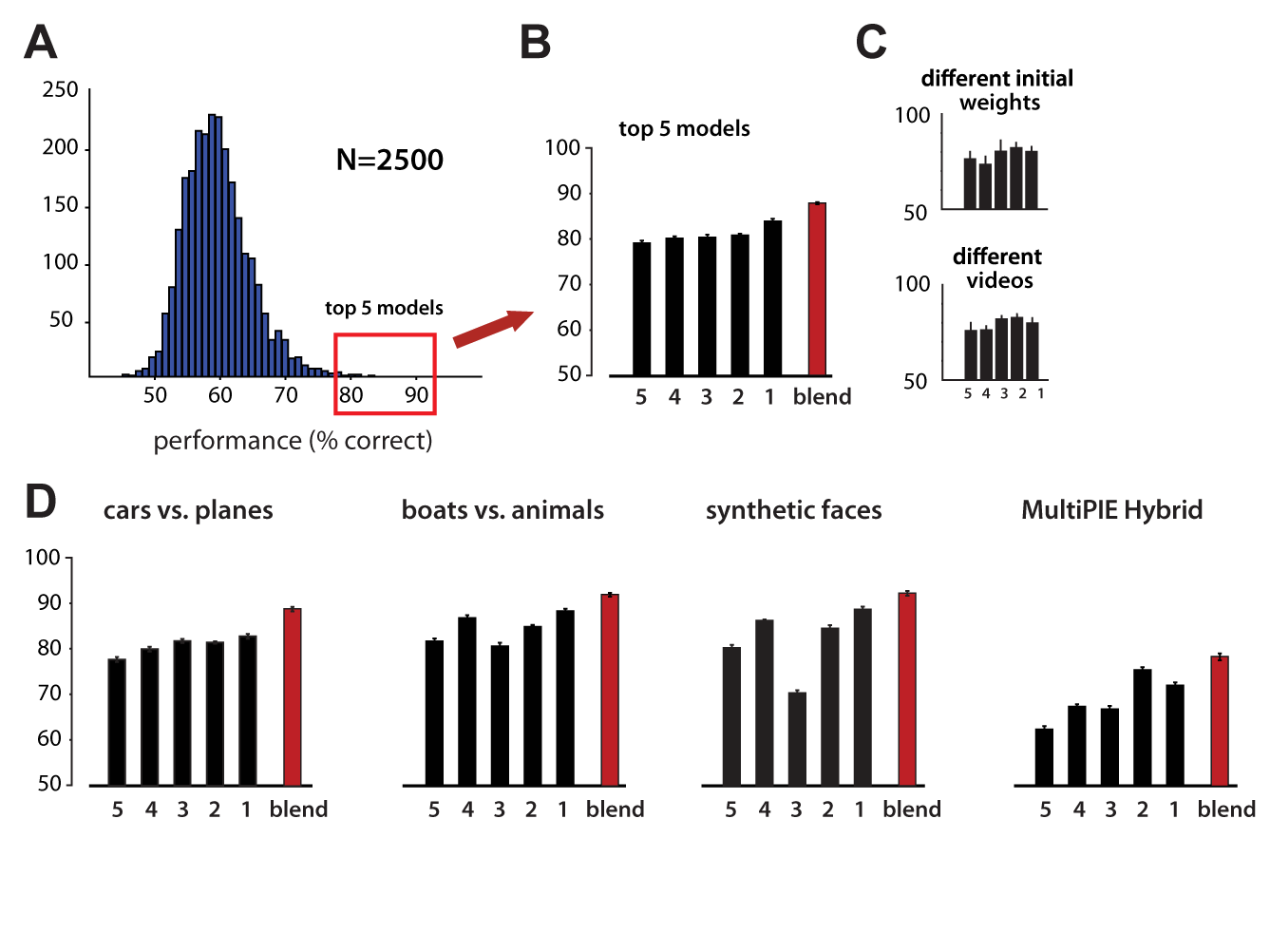

Supplement: Figure S8 — High-throughput screening and validation in the “Boats'” Petri Dish. Data are shown according to the same display convention set forth in the main paper. (A) Histogram of the performance of 2,500 models on the “Cars vs. Planes” screening task. The top five performing models were selected for further analysis. (B) Performance of the top five models (1–5). (C) Performance of the top five models when trained with a different random initialization of filter weights (top) or with a different set of video clips (bottom). (D) Performance of the top five models from the Screening Phase on a variety of other object recognition challenges. (0.22 MB TIF) [file pcbi.1000579.s008.tif]

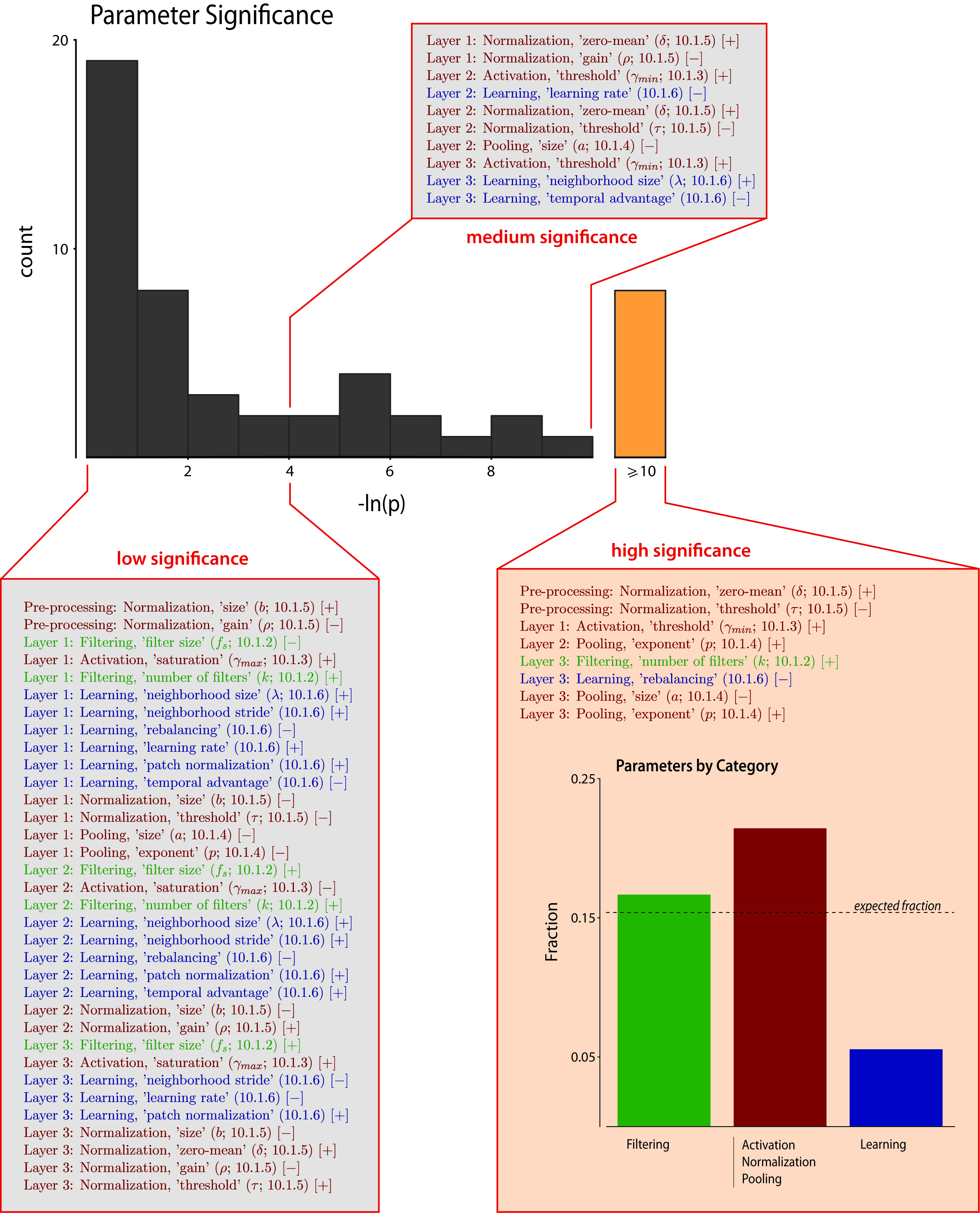

Supplement: Figure S9 — Linear regression analysis of relationship between parameter values and model performance. As a first-order analysis of the relationship between model parameters and model performance, we performed a linear regression analysis in which the values of each of the 52 parameters were included as predictors in a multiple linear regression analysis. Next, p-values were computed for the t statistic on each beta weight in the regression. A histogram of the negative natural log of the p-values is shown here, with the bin including significant p-values highlighted in orange (each count corresponds to one model parameter). For reference, the histogram is divided into three ranges (low-nonsignificant, medium-nonsignificant, and significant) and a listing of parameters included each significance range is printed below the histogram. Each parameter listing includes a 1) verbal description of the parameter, 2) its symbol according to the terminology in the Supplemental Methods, 3) the section number where it is referenced, and 4) whether it was positively (“+”) or negatively (“−”) correlated with performance. In addition, the parameters were divided into three rough conceptual groups and were color-coded accordingly: Filtering (green), Normalization/Activation/Pooling (red), and Learning (blue). Beneath the bin corresponding to significantly predictive parameters, a bar plot showing the fraction of each group found in the set of significant parameters. The expected fraction, if the parameters were distributed randomly, is shown as a dotted line. Activation/Normalization/Pooling parameters were slightly over-represented in the set of significantly-predictive parameters, but no group was found to be significantly over- or under-represented (p = 0.338; Fischer's exact test). (2.28 MB TIF) [file pcbi.1000579.s009.tif]

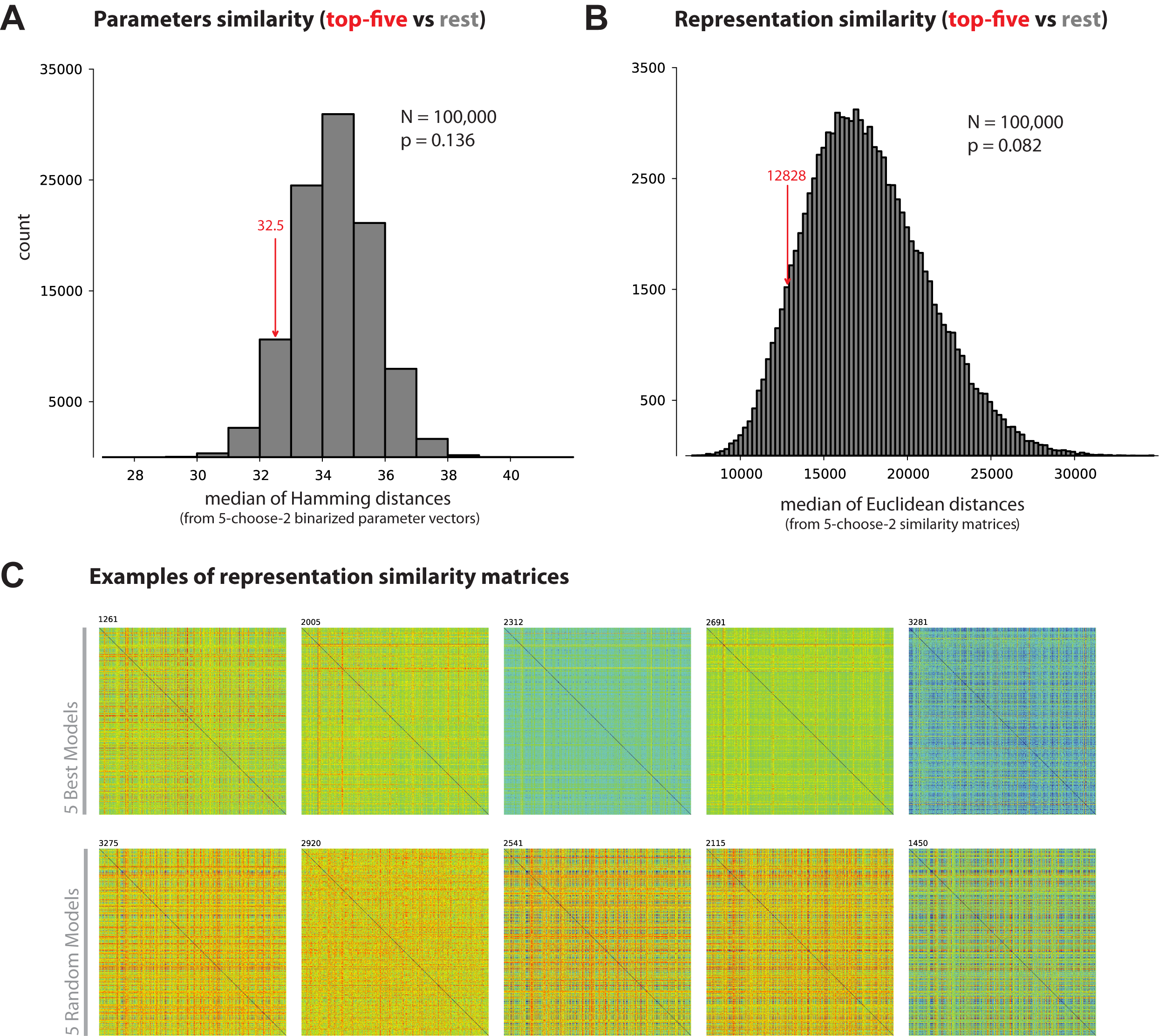

Supplement: Figure S10 — How similar are the top models? (A) Model similarity on the basis of parameter values (L0 or Hamming Distance). Each model is specified by a vector of 52 parameter values. As a first attempt at comparing models, we generated an expanded binary parameter vector in which every possible parameter/value combination was represented as a separate variable (e.g. a parameter ω that can take on values 3, 5, and 7 would be included in the expanded vector as three binary values [ω = 3], [ω = 5], and [ω = 7]). The Hamming distance distance between any two vectors can then serve as a metric of the similarity between any two models. In order to determine if the top five models taken from the “Law and Order” petri dish were more similar to each than would be expected of five randomly selected models, we computed the median pairwise Hamming distance between the top five models, and between a random sampling of 100,000 sets of five models taken from the remaining (non-top-five) models. The distribution of randomly selected model pairs is shown in (A), and the observed median distance amongst the top five models is indicated by an arrow. The top-five models tended to be more similar to one another than to a random selection of models from the full population, but this effect was not significant (p = 0.136; permutation test). (B) Model similarity on the basis of output (“Representation” similarity). As another way to compare model similarity, for each model we computed model output vectors for a selection of 600 images taken from the Screening task image sets. We then computed the L2 (Euclidean) distance matrix between these “re-represented” image vectors as a proxy for the structure of the output space of each model. A distance metric between any two models was then defined as the L2 distance between the unrolled upper-diagonal portion of the two models' similarity matrices (this distance metric is similar to the Frobenius norm). Finally, as in (A), the median distances between the t [file pcbi.1000579.s010.tif]
