# Supplementary material for: A High-Throughput Screening Approach to Discovering Good Forms of Biologically Inspired Visual Representation
Source: PLoS Comput Biol. 2009 Nov 26;5(11):e1000579. doi: 10.1371/journal.pcbi.1000579 (PMC2775908; doi:10.1371/journal.pcbi.1000579)
Supplement: Text S1 — Search Space of Candidate Models. (0.14 MB PDF) [file pcbi.1000579.s011.pdf]

A High-Throughput Screening Approach to Discovering Good Forms of Biologically-Inspired Visual Representation.

Nicolas Pinto, David Doukhan, James J. DiCarlo, David D. Cox

Text S1: Search Space of Candidate Models

Candidate models were composed of a hierarchy of three layers, with each layer including a cascade of linear and nonlinear operations that produce successively elaborated nonlinear feature-map representations of the original image. A diagram detailing the flow of operations is shown in Figure S2, and, for the purposes of notation, the cascade of operations is represented as follows:

$$\begin{aligned}
&Layer^0:\\
&\qquad \mathbf{Input} \xrightarrow{\textbf{Grayscale}} \xrightarrow{\textbf{Normalize}} \mathbf{N^0}\\
&Layer^1:\\
&\qquad \mathbf{N^0} \xrightarrow{\textbf{Filter}} \mathbf{F^1} \xrightarrow{\textbf{Activate}} \mathbf{A^1} \xrightarrow{\textbf{Pool}} \mathbf{P^1} \xrightarrow{\textbf{Normalize}} \mathbf{N^1}
\end{aligned}$$

and generally, for all $\ell \geq 1$:

$$\begin{aligned}
&Layer^\ell:\\
&\qquad \mathbf{N}^{\ell-1} \xrightarrow{\textbf{Filter}} \mathbf{F}^\ell \xrightarrow{\textbf{Activate}} \mathbf{A}^\ell \xrightarrow{\textbf{Pool}} \mathbf{P}^\ell \xrightarrow{\textbf{Normalize}} \mathbf{N}^\ell
\end{aligned}$$

Details of these steps along with the range of parameter values included in the random search space are described below. We varied 52 parameters (described below), with a total of 2.807930×10^{25} possible unique combinations of parameter values.

1 Input and Pre-processing

The input of the model was a 200×200 pixel image. In the pre-processing stage, referred to as $Layer^0$, this input was converted to grayscale and locally normalized:

$$\mathbf{N^0} = \mathbf{Normalize}(\mathbf{Grayscale}(\mathbf{Input})) \tag{1}$$

where the **Normalize** operation is described in detail below. Because this normalization is the final operation of each layer, in the following sections, we refer to $N^{\ell-1}$ as the input of each $Layer^{\ell>0}$ and N^{ℓ} as the output.

2 Linear Filtering

Description: The input $N^{\ell-1}$ of each subsequent layer (i.e. $Layer^{\ell}, \ell \in \{1,2,3\}$) was first linearly filtered using a bank of k^{ℓ} filters to produce a stack of k^{ℓ} feature maps, denoted F^{ℓ}. In a biologically-inspired context, this operation is analogous to the weighted integration of synaptic inputs, where each filter in the filterbank represents a different cell.

Definitions: The filtering operation for $Layer^{\ell}$ is denoted:

$$\mathbf{F}^{\ell} = \mathbf{Filter}(\mathbf{N^{\ell-1}}, \mathbf{\Phi}^{\ell}) \tag{2}$$

and produces a stack, F^{ℓ}, of k^{ℓ} feature maps, with each map, F_i^{ℓ}, given by:

$$F_i^{\ell} = N^{\ell-1} \otimes \Phi_i^{\ell} \qquad \forall i \in \{1, 2, \ldots, k^{\ell}\} \tag{3}$$

where $\otimes$ denotes a correlation of the output of the previous layer, $N^{\ell-1}$ with the filter Φ_i^{ℓ} (e.g. sliding along the first and second dimensions of $N^{\ell-1}$). Because each successive layer after $Layer^0$, is based on a stack of feature maps, $N^{\ell-1}$ is itself a stack of 2-dimensional feature maps. Thus the filters contained within Φ^{ℓ} are, in turn, 3-dimensional, with the their third dimension matching the number of filters (and therefore, the number of feature maps) from the previous layer (i.e. $k^{\ell-1}$).

Parameters:

- The filter shapes ${f_s}^{\ell} \times {f_s}^{\ell} \times {f_d}^{\ell}$ were chosen randomly with ${f_s}^{\ell} \in \{3,5,7,9\}$ and ${f_d}^{\ell} = k^{\ell-1}$.
- Depending on the layer ℓ considered, the number of filters k^{ℓ} was chosen randomly from the following lists:
  - In $Layer^1$, $k^1 \in \{16, 32, 64\}$
  - In $Layer^2$, $k^2 \in \{16, 32, 64, 128\}$
  - In $Layer^3$, $k^3 \in \{16, 32, 64, 128, 256\}$

All filters were initialized to random starting values, and their weights were then learned during the *Unsupervised Learning Phase* (described below; an example of a set of learned filterbanks from one model instance is shown in Figure S6).

3 Activation Function

Description: Filter outputs were subjected to threshold and saturation activation function, wherein output values were clipped to be within a parametrically defined range. This operation is analogous to the spontaneous activity thresholds and firing saturation levels observed in biological neurons.

Definitions: We define the activation function:

$$\mathbf{A}^{\ell} = \mathbf{Activate}(\mathbf{F}^{\ell}) \tag{4}$$

that clips the outputs of the filtering step, such that:

$$\mathbf{Activate}(\mathbf{x}) = \begin{cases} {\gamma_{max}}^{\ell} & \text{if } x > {\gamma_{max}}^{\ell} \\ {\gamma_{min}}^{\ell} & \text{if } x < {\gamma_{min}}^{\ell} \\ x & \text{otherwise} \end{cases} \tag{5}$$

Where the two parameters ${\gamma_{min}}^{\ell}$ and ${\gamma_{max}}^{\ell}$ control the threshold and saturation, respectively. Note that if both minimum and maximum threshold values are $-\infty$ and $+\infty$, the activation is linear (no output is clipped).

Parameters:

- ${\gamma_{min}}^{\ell}$ was randomly chosen to be $-\infty$ or 0
- ${\gamma_{max}}^{\ell}$ was randomly chosen to be 1 or $+\infty$

4 Pooling

Description: The activations of each filter within some neighboring region were then pooled together and the resulting outputs were spatially downsampled.

Definitions: We define the pooling function:

$$\mathbf{P}^\ell = \mathbf{Pool}(\mathbf{A}^\ell) \tag{6}$$

such that:

$$\mathbf{P}^\ell_\mathbf{i} = \mathbf{Downsample}_\alpha(\sqrt[p^\ell]{(A^\ell_i)^{p^\ell} \odot \mathbf{1}_{a^\ell \times a^\ell}}) \tag{7}$$

Where $\odot$ is the 2-dimensional correlation function with $\mathbf{1}_{a^\ell \times a^\ell}$ being an $a^\ell \times a^\ell$ matrix of ones (a^ℓ can be seen as the size of the pooling "neighborhood"). The variable p^ℓ controls the exponents in the pooling function.

Parameters:

- The stride parameter α was fixed to 2, resulting in a downsampling factor of 4.
- The size of the neighborhood a^ℓ was randomly chosen from $\{3, 5, 7, 9\}$.
- The exponent p^ℓ was randomly chosen from $\{1, 2, 10\}$.

Note that for $p^\ell = 1$, this is equivalent to blurring with a $a^\ell \times a^\ell$ boxcar filter. When $p^\ell = 2$ or $p^\ell = 10$ the output is the L^{p^ℓ}-norm [1].

5 Normalization

Description: As a final stage of processing within each layer, the output of the Pooling step were normalized by the activity of their neighbors within some radius (across space and across feature maps). Specifically, each response was divided by the magnitude of the vector of neighboring values if above a given threshold. This operation draws biological inspiration from the competitive interactions observed in natural neuronal systems (e.g. contrast gain control mechanisms in cortical area V1, and elsewhere [1, 2])

Definitions: We define the normalization function:

$$\mathbf{N}^\ell = \mathbf{Normalize}(\mathbf{P}^\ell) \tag{8}$$

such that:

$$N^\ell = \begin{cases} \rho^\ell \cdot C^\ell & \text{if } \rho^\ell \cdot \left|\left|C^\ell \otimes \mathbf{1}_{b^\ell \times b^\ell \times k^\ell}\right|\right|_2 < \tau^\ell \\ \frac{C^\ell}{\left|\left|C^\ell \otimes \mathbf{1}_{b^\ell \times b^\ell \times k^\ell}\right|\right|_2} & \text{otherwise} \end{cases} \tag{9}$$

[1]The L^{10}-norm produces outputs similar to a *max* operation (i.e. *softmax*).

with

$$C^\ell = P^\ell - \delta^\ell \cdot \frac{P^\ell \otimes \mathbf{1}_{b^\ell \times b^\ell \times k^\ell}}{b^\ell \cdot b^\ell \cdot k^\ell} \tag{10}$$

Where $\delta^\ell \in \{0,1\}$, $\otimes$ is a 3-dimensional correlation over the "valid" domain (i.e. sliding over the first two dimensions only), and $\mathbf{1}_{b^\ell \times b^\ell \times k^\ell}$ is a $b^\ell \times b^\ell \times k^\ell$ array full of ones. b^ℓ can be seen as the normalization "neighborhood" and δ^ℓ controls if this neighborhood is centered (i.e. subtracting the mean of the vector of neighboring values) before divisive normalization. ρ^ℓ is a "magnitude gain" parameter and τ^ℓ is a threshold parameter below which no divisive normalization occurs.

Parameters:

- The size b^ℓ of the neighborhood region was randomly chosen from $\{3, 5, 7, 9\}$.
- The δ^ℓ parameter was chosen from $\{0, 1\}$.
- The vector of neighboring values could also be stretched by gain values $\rho^\ell \in \{10^{-1}, 10^0, 10^1\}$. Note that when $\rho^\ell = 10^0 = 1$, no gain is applied.
- The threshold value τ^ℓ was randomly chosen from $\{10^{-1}, 10^0, 10^1\}$.

6 Final model output dimensionality

The output dimensionality of each candidate model was determined by the number of filters in the final layer, and the x-y "footprint" of the layer (which, in turn, depends on the subsampling at each previous layer). In the model space explored here, the possible output dimensionalities ranged from 256 to 73,984.

7 Unsupervised Learning

Description: During the *Unsupervised Learning Phase*, filter weights are learned from input video sequences. This procedure bears similarity to non-parametric density estimation, e.g. online K-means clustering. The algorithm for this phase additionally contains simple mechanisms for taking advantage of temporal information in a video sequence, and thus *Unsupervised Learning* was conducted on sequences of video frames. In this work, 15,000 video frames were used.

Definitions: For each incoming video frame, an output for each filter at each location was computed, and a "winning" filter Φ^{ℓ}_{winner} was selected:

$$winner = \arg\max_i (F^{\ell}_i) \tag{11}$$

This winning filter was adapted to the input, by adding the corresponding input patch, times a fixed learning rate λ, to the filter weights:

$$\Phi^{\ell}_{winner}{}' = (1 - \lambda^{\ell}) \cdot \Phi^{\ell}_{winner} + \lambda^{\ell} \cdot patch \tag{12}$$

The resulting updated filter was then re-normalized to zero-mean and unit-length:

$$\Phi^{\ell}_{winner}{}'' = \frac{\Phi^{\ell}_{winner}{}' - \langle \Phi^{\ell}_{winner}{}' \rangle}{||\Phi^{\ell}_{winner}{}' - \langle \Phi^{\ell}_{winner}{}' \rangle||_2} \tag{13}$$

Where $\langle \Phi^{\ell}_{winner}{}' \rangle$ represents the mean of the winner's weights and $\Phi^{\ell}_{winner}{}''$ is the filter carried forward into the next learning iteration.

The incoming patch could be normalized (i.e. $||patch||_2 = 1$), or not, under parametric control, and multiple patches could enter into one "round" of competition at the same time (e.g. filter stack outputs corresponding to multiple patches could be evaluated, and the largest output across all patches could decide the winner). The selection of the number of patches simultaneously competing was governed by the *Competition Neighborhood Size* and *Competition Neighborhood Stride* parameters, which served tile a set of competing filter stacks across the input.

Parameters:

- *Learning rate* parameter $\lambda^{\ell} \in \{10^{-4}, 10^{-3}, 10^{-2}\}$
- *Patch Normalization*: normalize *patch* to unit-length, or do not normalize (2 choices)
- *Competition Neighborhood Size* $\in \{1, 3, 5, 7, 9\}$
- *Competition Neighborhood Stride* $\in \{1, 3, 5, 7, 9\}$
- *"Rebalancing"*: if the relative winning ratio [2] of a given filter Φ^{ℓ}_i is less than {1%, 10% or 50%} (3 choices), its weights are reinitialized to the values of the most-winning filter plus a random jitter. This prevents filters from never winning.

[2] the number of times Φ^{ℓ}_i won multiplied by the number of filters, divided by the running count of completed updates

- *"Temporal Advantage"* (or *"trace"*, see also [3, 4, 5, 6] for variants): the output score of the last-winning filter is multiplied by {1, 2 or 4} (3 choices) prior to determining which filter "wins." A value of 1 is the equivalent of no advantage; a value of 2 doubles the effective output of the filter for the purposes of competition, biasing it to win again.

8 Classification during Screening and Validation Phases

During the *Screening* and *Validation Phases*, the representations generated during the *Unsupervised Learning Phase* were evaluated in a variety of object recognition tasks (see main text). This *Classification Phase* consisted of the following steps, with *fixed parameters* across all model instantiations:

- A random sampling of up to 5,000 outputs from the full representation were taken (to accelerate processing).
- Dimensionality was further reduced by PCA (using training data only, keeping the full eigensubspace projection, i.e. as many dimensions as training examples).
- A linear SVM (using the libsvm[3] solver, with regularization parameter $C = 10$) was used with a 10-trial random subsampling cross-validation scheme (150 training and 150 testing examples).

9 Random Exploration

Note that the parameters and parameter ranges described here are clearly not the most comprehensive search space; rather they represent a starting point intended to demonstrate the utility of the overarching approach. While a brute force search procedure was used here, other more elaborate optimization schemes (e.g. evolutionary algorithms [7]) could also be used.

* * *

[3] http://www.csie.ntu.edu.tw/~cjlin/libsvm

References

1. Geisler WS, Albrecht DG (1992) Cortical neurons: isolation of contrast gain control. Vision Research 32: 1409–10.

2. Rolls ET, Deco G (2002) Computational neuroscience of vision. Oxford University Press New York.

3. Foldiak P (1991) Learning Invariance from Transformation Sequences. Neural Computation 3: 194–200.

4. Rolls ET, Milward T (2000) A Model of Invariant Object Recognition in the Visual System: Learning Rules, Activation Functions, Lateral Inhibition, and Information-Based Performance Measures. Neural Computation 12: 2547–2572.

5. Einhäuser W, Hipp J, Eggert J, Körner E, König P (2005) Learning viewpoint invariant object representations using a temporal coherence principle. Biological Cybernetics 93: 79–90.

6. Franzius M, Wilbert N, Wiskott L (2008) Invariant Object Recognition with Slow Feature Analysis. Proc 18th Intl Conf on Artificial Neural Networks, ICANN'08, Prague .

7. Deb K (2001) Multi-Objective Optimization Using Evolutionary Algorithms. Wiley.
