# Supplementary material for: A High-Throughput Screening Approach to Discovering Good Forms of Biologically Inspired Visual Representation
Source: PLoS Comput Biol. 2009 Nov 26;5(11):e1000579. doi: 10.1371/journal.pcbi.1000579 (PMC2775908; doi:10.1371/journal.pcbi.1000579)
Supplement: Text S2 — Technical Details of the Computational Framework. (0.08 MB PDF) [file pcbi.1000579.s012.pdf]

A High-Throughput Screening Approach to Discovering Good Forms of Biologically-Inspired Visual Representation.

Nicolas Pinto, David Doukhan, James J. DiCarlo, David D. Cox

Text S2: Technical Details of the Computational Framework

The high-throughput search described in this paper takes advantage of multiple levels of parallelism, from coarse to fine-grained. Roughly speaking, fine-grained parallelism is exploited by allocating one core (of which modern graphics hardware have many; and their number is exponentially growing over the years) to one or more virtual neurons, while coarse-scale parallelism is achieved by allocating one model instantiation to each of many multi-core pools (i.e. CPUs, Cell Processors, GPUs). Because we evaluated thousands of model instantiations, it was straightforward to spread these evaluations across a cluster of GPU-enabled nodes, with the throughput of each node maximized by taking full advantage of fine-grained parallelism.

In practice, as a general rule in modern high-performance computing, the level of speed-up that is achievable depends more fundamentally on the ability to bring relevant memory fetches to the parallel arithmetic processing units, than on the number of these units per se. For this reason, stream processing architectures contain special mechanisms for explicit manipulation of fast local caches (e.g. the Local Stores on the Cell processor and Shared Memory on NVIDIA GPUs). Importantly for maximizing the usage of the parallel resources in these processors, some of the largest computational bottlenecks present in our model class (e.g. filtering operations), lend themselves to the usage of such caches by loading small tiles of data into local caches. More broadly, because each layer of each model maintains a notion of x-y space, and because most of the operations operate over spatially local regions (e.g. normalization occurs within a spatially-restricted neighborhood), our coarse-to-fine-grain parallelism can be exploited throughout a large portion of our model implementation.

The use of commodity graphics hardware has drastically reduced the cost

of a scientific exploration of this scale (see Table 1 and Supplemental Figure S1), but writing reliable code for these multi-core platforms can be a demanding task. On one hand, scientific software can be difficult to handle because of constantly changing requirements. On the other hand, these architectures are advancing at a very rapid pace and we have experienced three different paradigms in three years (i.e. programming GPUs with graphics primitives in 2006, programming the PlayStation 3 using low-level Cell intrinsics in 2007 and programming GPUs with compute primitives in 2008; see below). To overcome these difficulties, we combined careful engineering, high-level languages (such as Python[1] and its numerous scientific bindings[2],[3]) and template meta-programming techniques. Inspired by automatically-tuned scientific libraries such as the Automatically Tuned Linear Algebra Software (ATLAS[4]) or the Fastest Fourier Transform in the West library (FFTW[5]), we found that empirical optimization through automatic run-time code generation was a useful way to abstract the low-level details away from the end-user. Integrating these heterogeneous technologies in our large-scale computational software shows us that it is possible to achieve a favorable balance between ease-of-use, ease-of-programming and peak computing speed.

An extensive description on how we used these techniques is well beyond the scope of this paper, however, we highlight a few important high-level points here:

- *Meta-programming and combining high-level with low-level languages*

  In our implementation, each core operation (see Text S1) has two levels of abstraction. The high-level abstraction is designed for the user and is written in a high-level language (we used Python). The low-level abstraction is designed to achieve maximum throughput on heterogeneous hardware and as a consequence it must be able to handle low-level languages and "close to the metal" code optimization techniques (e.g. involving assembly) if needed. The interface between the two abstractions is a templating engine (we used Cheetah[6]) that is responsible for dynamically generating optimized low-level code at runtime, many specialized versions of which are compiled and auto-tuned prior to running a given model simulation. Such an approach is equivalent to "just-in-time" (JIT[7]) compilation techniques used elsewhere for portability and

[1]http://www.python.org
[2]http://numpy.scipy.org
[3]http://www.scipy.org
[4]http://www.netlib.org/atlas
[5]http://www.fftw.org
[6]http://www.cheetahtemplate.org
[7]http://en.wikipedia.org/wiki/Just-in-time_compilation

dynamic specialization[8].

The primary goal of the developer is to come up with various optimization strategies that instrumentalize low-level code and manipulate it from a high-level language (using templates). These strategies may involve loop unrolling[9], software pipelining[10], register pressure[11], communication and computation load distribution (aka "latency hiding"), to name just a few.

Producing a large number of hand-tuned implementations, corresponding to optimized lower-level code across a range of implementations would be impractically time-consuming. A meta-programming approach circumvents this difficulty by producing code that can itself generate a variety of specialized compiled versions under parametric control. This large number of candidate implementations of the meta-program can be empirically tested to find which is the fatest (see *Auto-tuning* below)

It is important to note that the high-level language must be mature and general enough to allow a seamless interaction between all the components of the system, from the distributed job system and its database to its template meta-programming capabilities and its interaction with other (low-level) languages. The Python programming language was a natural choice as it is often referred as a versatile "glue" language (i.e. used to connect software components of different levels together), and allows quick prototyping and experimentation.

While MATLAB, by itself, does not support easy meta-programming on GPUs, commercial companions to MATLAB like AccelerEyes's Jacket[12] could potentially enable some of the gains necessary for our approach. However, such solutions typically do not necessarily achieve the full performance of stream processing hardware [13].

- *Auto-tuning*

  To auto-tune our instrumentalized (i.e. templated) code, we used the simplest approach: random search on a coarse grid. Using this simple approach, we achieved comfortable speed-ups, and thus we did not

[8] http://psyco.sourceforge.net
[9] http://en.wikipedia.org/wiki/Loop_unwinding
[10] http://en.wikipedia.org/wiki/Software_pipelining
[11] http://en.wikipedia.org/wiki/Register_allocation
[12] http://www.accelereyes.com
[13] http://www.nvidia.com/object/matlab_acceleration.html

explore more complex schemes before launching the experiments presented in this study. In the future, we plan to investigate the use of machine learning techniques to auto-tune the code, an approach recently undertaken by IBM's Milepost GCC[14].

- *Use of specialized extensions and libraries*

  Our PlayStation 3 implementation was created using tools provided in IBM's Cell SDK (Software Development Kit[15]) were mature and comprehensive (e.g. availability of a simulator, profiler, debugger, etc.), interfaced using ctypes[16] from the Python standard library. These tools allow one to program primarily in C (or in a language that can bind to an underlying C implementation), but require specialized knowledge of the architecture of the Cell processor in order to achieve high levels of performance.

  Our NVIDIA GPU implementation was created using NVIDIA's CUDA programming model (an augmented superset of C), managed via Py-CUDA [17] and python-cuda [18], Python libraries that bind to the underlying NVIDIA CUDA libraries. Meta programs were created using the Cheeath template library (see above) that would emit specialized CUDA code which was compiled on the fly and run on the GPU.

  While the efforts described here relied on vendor-specific software development kits (which arguably imposes a significant barrier-to-entry for developer scientists), efforts are underway in industry to provide a unified programming model and tool set for developing applications of the sort presented here. In particular, the lack of general-purpose programming standard for heteogeneous systems was recently addressed through the introduction of OpenCL[19] (Open Computing Language) by the Khronos Group. We anticipate future work to utilize these tools will enable us to target more platforms, and will ease the cost of incorporating ideas of the sort presented here into the work of other groups.

[14] http://www.milepost.eu
[15] http://www.ibm.com/developerworks/power/cell
[16] http://docs.python.org/library/ctypes.html
[17] http://mathema.tician.de/software/pycuda
[18] http://github.com/npinto/python-cuda
[19] http://www.khronos.org/opencl
