# Supplementary material for: A High-Throughput Screening Approach to Discovering Good Forms of Biologically Inspired Visual Representation
Source: PLoS Comput Biol. 2009 Nov 26;5(11):e1000579. doi: 10.1371/journal.pcbi.1000579 (PMC2775908; doi:10.1371/journal.pcbi.1000579)
Supplement: Text S3 — First-Order Analyses of Model Parameters and Behavior. (0.05 MB PDF) [file pcbi.1000579.s013.pdf]

A High-Throughput Screening Approach to Discovering Good Forms of Biologically-Inspired Visual Representation.

Nicolas Pinto, David Doukhan, James J. DiCarlo, David D. Cox

Text S3: First-Order Analyses of Model Parameters and Behavior

The results presented in the main text show that the five best model instantiations found by a screening procedure are well-suited to a variety of object recognition tasks, but they do not speak to *how* these models achieve their performance. While fully answering this question is beyond the scope of the present paper, as a first step in understanding model performance, we asked a series of first-order questions about the relationship between model parameters and performance. These analyses are in no way intended to be definitive; rather, they primarily suggest directions and challenges for future experiments.

First, we asked which (if any) of the parameters were predictive of model performance, using simple linear regression. While complex interdependencies between parameters can (and almost certainly do) exist, linear regression provides a first-order tool to identify parameters that are especially important to performance. Significance values for individual parameters are shown in a histogram in Figure S9. A handful of parameters were found to be significantly predictive of model performance. To determine if a particular category of model parameters were more important than any other, we divided the parameters into three groups: linear filter parameters, normalization/activation/pooling nonlinear parameters, and learning parameters. We found that normalization/activation/pooling parameters shared a trend toward being over-represented in the set of significantly predictive parameters, but that the distribution of significant parameters from each of these three categories were not significantly different than would be predicted by chance ($p = 0.338$; Fisher's exact test).

Another reasonable first-order question to ask is whether the top models are somehow similar to one another. In this context, similarity might be assessed along a number of axes. One possibility is to simply compare the parameter values for the best models, to see if they share more parameter settings in common with each other than one would expect by chance. To do this, an expanded binary parameter vector was first created in which each parameter value combination was included as a binary element (e.g. if a parameter

ω could take of values 3, 5, and 7, three binary values $[\omega = 3]$, $[\omega = 5]$, and $[\omega = 7]$ were generated for each model). The Hamming distance was then computed between these vectors to assess the similarity between models. To determine whether the top five models were more similar to each other than to the population of models, we computed the median pairwise Hamming distance among the top five, and among randomly chosen sets of five models ($N = 100,000$) taken from the remaining (non-top-five) models (Figure S10a). By this measure, the median distance between the top five trended toward higher than expected similarity but was not found to be significantly different from the median distance over the full population of models ($p = 0.136$; permutation test). Thus, at least by this simple measure, we could find no evidence that the best models were any more similar to each other than would be expected by chance. Attempts to compare parameter vector using ℓ_1 and ℓ_2 distances also failed to find any increased similarity amongst the best models, though these analyses are intrinsically difficult to interpret, as it is unclear how to scale one parameter relative to another.

Another approach to comparing models is to compare the structure of the space of their outputs. That is, for a given set of images, do the best models somehow transform these images in a similar way? To explore this issue, we transformed 600 images from the screening set for each model, and then formed the similarity (Euclidean distance) matrix for the set of transformed image vectors. We then computed the Euclidean distance between the upper triangular part of these symmetric matrices (similar to the Frobenius distance) to assess their similarity. As before, we computed distributions of pairwise distances within the top five models ($N = \binom{5}{2} = 10$), and in the random sampling from the full population ($N = 10,000$) in order to test whether the top five models were more similar to each other than would be expected from random draws of five models (Figure S10b and S10c). We found that the similarity matrices of the top five models tended to be more similar to each other, but that this effect was not significant ($p = 0.082$; permutation test).
